# Integrated Genomic and Functional Analyses Identify *FaNAC6* as a Candidate Regulator of Drought and Salinity Responses in Strawberry (*Fragaria* × *ananassa*)

**DOI:** 10.3390/ijms27167352

**Published:** 2026-08-17

**Authors:** Facundo Spadoni-Revol, María Dolores Moreno-Recio, Sara Aguado-Delgado, Sara Posé, José A. Mercado, José Luis Caballero, Enriqueta Moyano, Francisco J. Molina-Hidalgo, Josefina P. Fernández-Moreno, Francisco J. Ruiz-Gómez, Juan Muñoz-Blanco, Rosario Blanco-Portales

**Affiliations:** 1 Departamento de Bioquímica y Biología Molecular, Universidad de Córdoba, Edificio Severo Ochoa (C6), Campus Universitario de Rabanales, 14071 Cordoba, Spain; bb2spref@uco.es (F.S.-R.); b42morem@uco.es (M.D.M.-R.); b62agdes@uco.es (S.A.-D.); bb1carej@uco.es (J.L.C.); bb2mocae@uco.es (E.M.); b52mohif@uco.es (F.J.M.-H.); jpfernadez@uco.es (J.P.F.-M.); 2 Instituto de Hortofruticultura Subtropical y Mediterránea ‘La Mayora’ (IHSM-UMA-CSIC), Departamento de Botánica y Fisiología Vegetal, Universidad de Málaga, 29071 Málaga, Spain; sarapose@uma.es (S.P.); mercado@uma.es (J.A.M.); 3 Instituto Interuniversitario del Sistema Tierra de Andalucía (IISTA), Universidad de Córdoba, Campus de Rabanales, Edificio Leonardo Da Vinci, Crta. N-IV km. 396, 14071 Cordoba, Spain; g72rugof@uco.es

**Keywords:** abiotic stress, drought, *Fragaria* × *ananassa*, NAC transcription factors (TFs), salinity, strawberry, water-use efficiency (WUE)

## Abstract

NAC proteins are a large family of plant transcription factors that play a key role in growth, development and responses to abiotic stress (drought, salinity). Various genomic studies across several species have identified *NAC* genes with specific responses to cold, drought and salinity. This study conducts a comprehensive genomic and transcriptomic analysis of *FaNAC* genes in strawberry (*Fragaria* × *ananassa*) to elucidate their role in response to drought and salinity. Using RNA-seq and validation by RT-qPCR, thirty-five *FaNAC* genes were identified that showed differential expression under both stress conditions. Their expression profiles were highly specific to tissue and stimulus type: some were induced in leaves and roots under both conditions, whilst others showed restricted induction. This suggests a specialised regulatory network rather than a uniform response. Phylogenetically, the *FaNAC* genes showed high homology with *Arabidopsis* orthologues and conserved collinearity between subgenomes. The promoters contained cis elements associated with hormones and stress, particularly ABA and MeJA. *FaNAC6* was selected for its strong induction in leaves and roots in response to drought and salinity, as well as under oxidative stress and ABA. Its overexpression in *Nicotiana benthamiana* increased stress tolerance, improving photosynthesis and water-use efficiency, and was associated with the upregulation of genes involved in photosystems, electron transport and carbon fixation. Overall, *FaNAC6* emerges as a promising candidate for further evaluation in strawberry breeding strategies to improve drought and salinity resilience.

## 1. Introduction

Adverse environmental conditions (drought, low temperatures, excess heavy metals, high salinity, or nutrient deficiency) affect plant physiology and development and negatively impact the yield of many crops of agronomic interest [1]. Specifically, the effects of drought and salinity stress on crop production pose a significant economic and social challenge [2,3,4]. These stresses have been shown to limit photosynthesis, decrease germination and growth, and drastically influence the hormonal balance of the affected plant [5,6]. Consequently, in cases of dehydration stress, a signaling cascade is initiated that culminates in the generation of reactive oxygen species (ROS), which exert harmful effects on macromolecules and cellular function [6,7]. Conversely, saline stress has been shown to induce ionic imbalance, osmotic disturbances, and the accumulation of toxic substances, particularly through the excessive generation of reactive oxygen species (ROS) [6,8]. Strawberries are highly sensitive to water scarcity in the soil. In fact, the plant’s first response to drought is the inhibition of its growth and a reduction in its productivity [9,10]. Furthermore, various morphological changes occur, such as the induction of vegetative development due to increased nutrient transport to the aerial parts of the plant at the expense of the fruit. This results in a reduction in fruit size and a change in its flavor [9]. Similarly, it has been confirmed that sugar metabolism and carbohydrate transport are significantly affected by drought [9,10]. A study conducted with fifteen strawberry varieties subjected to water stress for two seasons demonstrated that drought significantly reduces the relative water and chlorophyll content and increases the carbohydrate content in the leaves [10]. Similarly, salinity has been shown to affect strawberry plant productivity, reducing the number and size of leaves, shoot weight, and the number of branch crowns [11]. In any case, when the plant receives a stress signal through membrane receptors, this stimulus is transformed into an intracellular signal that triggers a cascade of signals involving ROS, calcium ions (Ca^2+^), and phytohormones, such as abscisic acid (ABA). These act as second messengers, activating or repressing the binding of certain transcription factors (TFs) to stress-response genes and positively or negatively stimulating their transcription [3,6,12]. It is evident that TFs play a fundamental role in regulating the plant’s response to abiotic stresses, such as drought and salinity, by binding to specific cis-regulatory elements in the promoter regions of their target genes [6,13]. 

The NAC TFs (NAM-ATAF-CUC) represent one of the most abundant and specific regulatory families in plants. They are notable for their involvement in controlling responses to abiotic stress, disease resistance, ROS homeostasis, and regulating plant development [3,14,15,16,17,18,19]. Numerous studies have shown that *NAC* genes play a significant role in drought and salinity tolerance and response in various plants, including rice [20], *Arabidopsis* [21], wheat [22], peanut [23], soybean [24], and tomato [25]. Overexpression of *ANAC055*, *ANAC072*, *ANAC019*, *ZmNAC55*, and *PaNAC57* in *Arabidopsis* has been shown to result in increased drought tolerance [26,27,28]; similarly, overexpression of *TaNAC47* from wheat has been shown to increase ABA sensitivity in transformed *Arabidopsis* plants and increase the concentration of soluble sugars and osmoprotective compounds such as proline [29]. In rice, *OsNAC17* upregulates the expression of numerous genes in the lignin biosynthesis pathway, thereby promoting lignin accumulation in leaves and roots and enhancing drought tolerance [30]. Also in rice, OsNAC023 has been observed to interact with OsREM1.5 under conditions of water stress, subsequently translocating to the nucleus to activate pathways associated with the drought/heat response [31]. Furthermore, several studies on the overexpression of TF OsNACs, such as SNAC1 [32], OsNAC5 [33], OsNAC6 [34] and ONAC022 [35], have demonstrated a significant increase in the tolerance of transgenic rice plants to drought and salinity. For example, the overexpression of *SNAC1* enhanced the plant’s capacity to withstand severe water stress by modulating stomatal closure [36], and overexpression of *ONAC022* led the accumulation of high concentrations of proline and soluble sugars, as well as the induction of root system development in transgenic plants, through an ABA-mediated pathway, to promote water uptake and salinity tolerance [35]. In a similar manner, the overexpression of JUNGBRUNNEN1 in *Solanum lycopersicum* resulted in enhanced drought tolerance [37], as did the overexpression of *SbSNAC1* from sorghum in *A. thaliana* seedlings [38]. Similarly, overexpression of *ThNAC4* increased salinity tolerance in *Tamarix* and *Arabidopsis* [39]. Moreover, overexpressing the *ZmSNAC1* gene of maize led to a substantial enhancement in the ability to withstand elevated levels of salt and drought in transgenic *Arabidopsis* plants [40]. The same behavior was also observed in experiments involving the overexpression of *GmNAC20* in rice [41] or *GmNAC06* in soybean [42]. Specifically, the overexpression of *GmNAC06*, whose expression is induced by NaCl, induced the accumulation of proline and glycine betaine in transgenic *Arabidopsis* plants to reduce the negative effect of ROS, and balanced the Na^+^/K^+^ ratio in their roots to maintain ionic homeostasis in this tissue under saline stress conditions [42]. 

In strawberry (*Fragaria* spp.), studies have also been conducted on the role of *NAC* genes, mainly in relation to development, ripening, and post-harvest fruit quality [43,44,45,46,47]. However, there is evidence to suggest that *NAC* genes are directly involved in the strawberry plant’s response to salinity and/or water deficit conditions. In *Fragaria vesca*, a total of 37 *FvNAC* genes were found, and a subset of them showed significant differential expression in response to salinity, drought, low temperatures, and biotic stress, probably via ABA-dependent pathways and antioxidant mechanisms [48]. Similarly, *FaNAC2* overexpression in *Nicotiana benthamiana* has been shown to increase the expression of key genes in the proline biosynthesis pathway while downregulating those in the catabolic pathway [49]. Additionally, ABA biosynthesis gene expression was promoted [49]. Similar behavior was observed in transgenic *Arabidopsis* plants overexpressing *FvNAC29*, which increased their tolerance to salinity and low temperatures through increased concentrations of chlorophyll and proline, increased antioxidant enzyme activity and the regulation of *AtRD29a*, *AtCCA1*, *AtP5CS1* and *AtSnRK2.4* expression levels, all of these genes related to the ABA pathway [50]. In addition, a study of the drought response of *F. nilgerrensis*, based on an integrated analysis of DNA methylation, transcriptome, and physiological traits under drought stress conditions, revealed the activation of 19 *NAC* family genes with altered methylation profiles. This suggests an interaction between these TFs and epigenetic control mechanisms in adapting to water deficits [51].

In this study, a group of *FaNAC* genes related to the strawberry plant’s response to drought and salinity was identified. To this end, a transcriptomic study was conducted using RNA-seq on strawberry leaves and roots that were subjected to severe water stress and high salinity. Using bioinformatic tools, a phylogenetic analysis of the identified *FaNAC* genes was performed, together with a study of their collinearity and the identification of their conserved domains. An in silico analysis of these *FaNAC* gene promoters was also carried out to identify the cis-regulatory elements present in them, and the expression patterns of these genes under water and salt stress were investigated, allowing us to suggest a regulation model for these genes. Furthermore, the results obtained have also allowed us to identify the *FaNAC6* gene as a key gene in the response of strawberry plants to both drought and salinity, showing that its overexpression increases the drought and salinity resilience of transgenic *Nicotiana* plants. Therefore, the identification and characterization of these genes, and more specifically *FaNAC6*, will facilitate the identification of potential molecular targets to improve the resilience of strawberry plants to drought and salinity.

## 2. Results and Discussion

### 2.1. Identification and Analysis of Expression Patterns of Strawberry NAC Genes That Vary Their Expression in Response to Drought and Salinity

**Figure 1:**
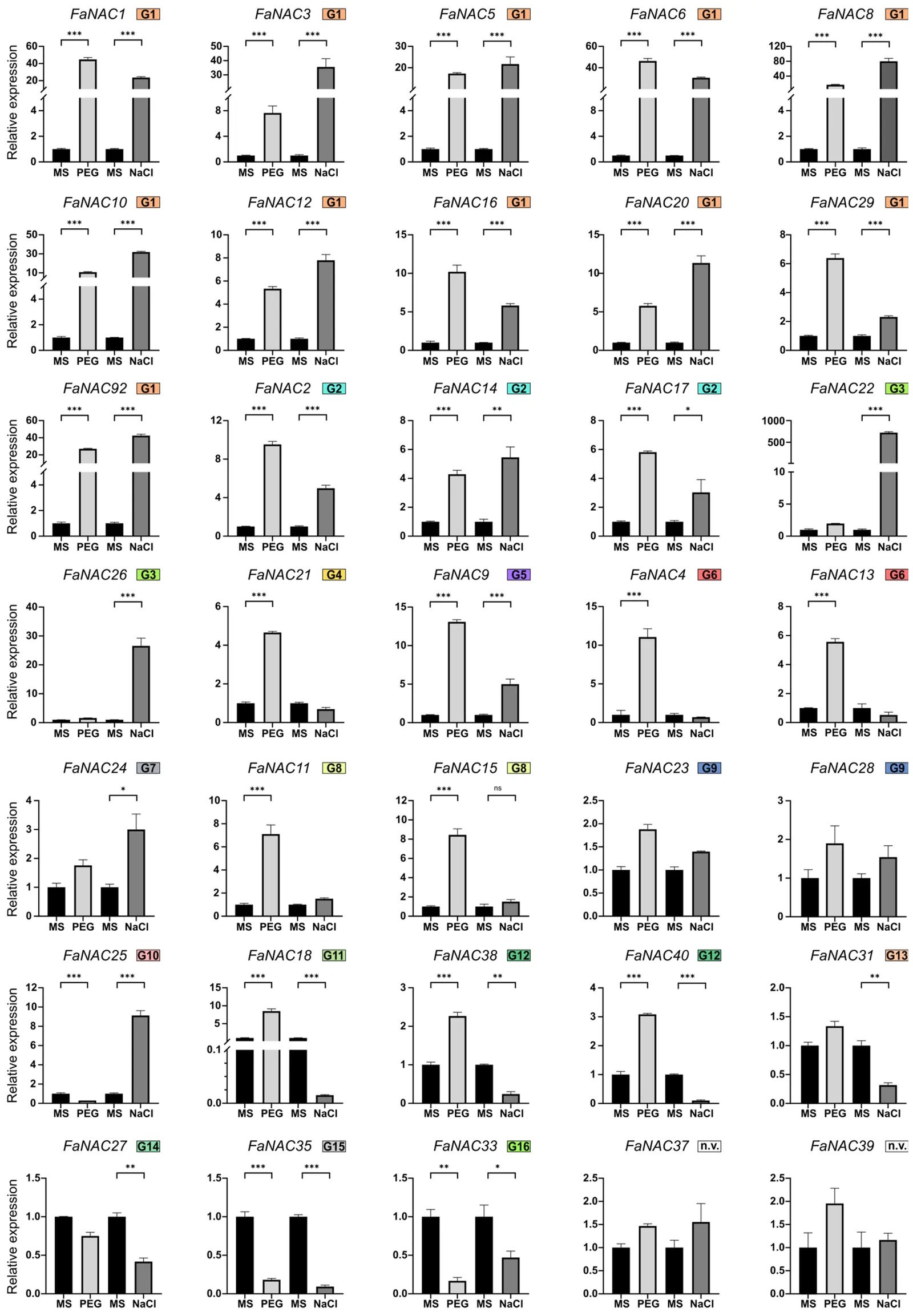
Expression pattern determined by RT-qPCR for *FaNAC* genes showing differential expression in leaves subjected to water stress (20% PEG) and salt stress (200 mM NaCl), as identified by RNA-seq. The data represent the mean of three independent experiments. MS: control plants; PEG and NaCl: treated plants. Asterisks indicate significant differences between control samples and stressed samples (* *p* ≤ 0.5; ** *p* ≤ 0.05; *** *p* ≤ 0.005). G1–16: different expression groups.

**Figure 2:**
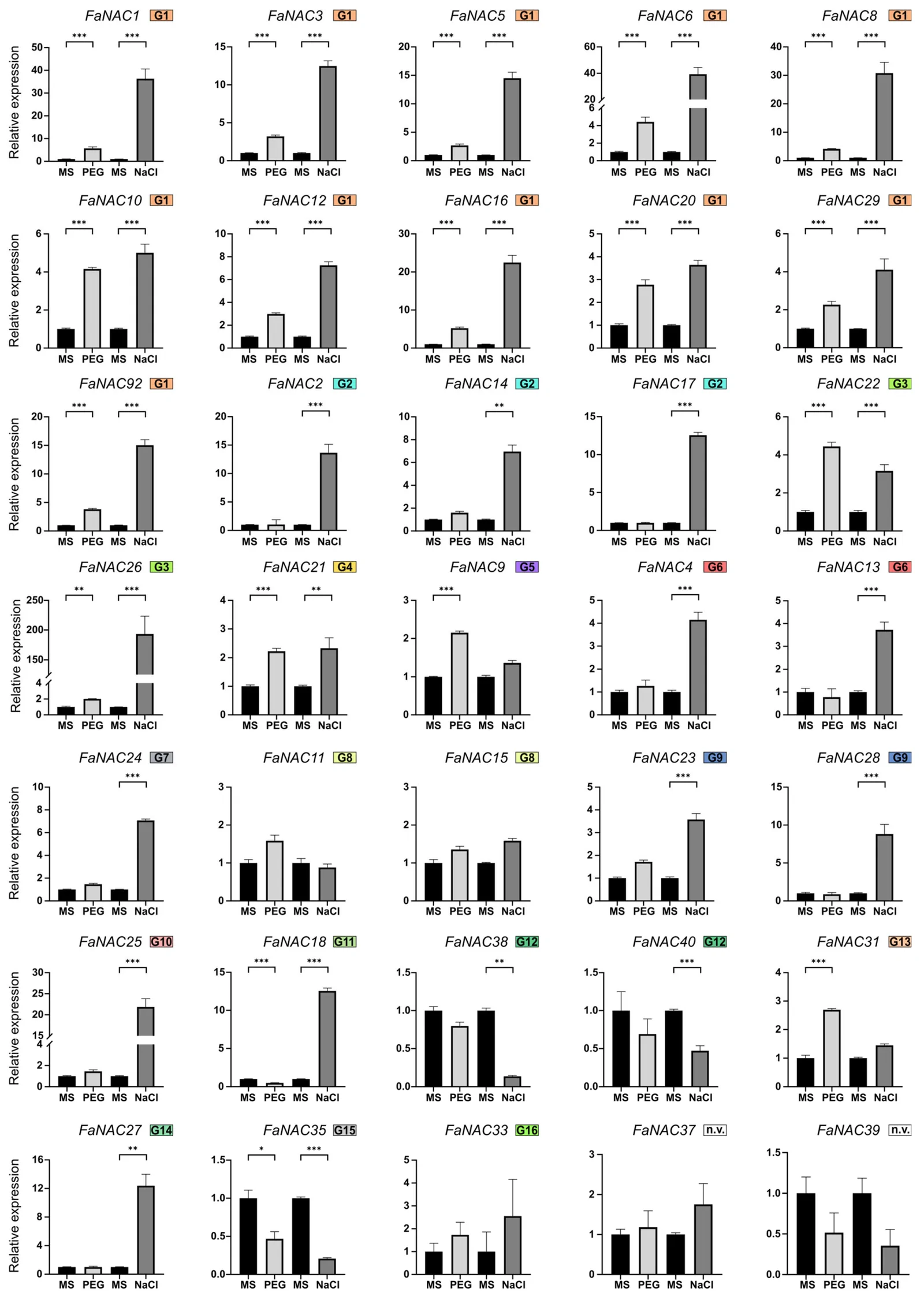
Expression pattern determined by RT-qPCR for *FaNAC* genes showing differential expression in roots subjected to water stress (20% PEG) and salt stress (200 mM NaCl), as identified by RNA-seq. The data represent the mean of three independent experiments. MS: control plants; PEG and NaCl: treated plants. Asterisks indicate significant differences between control samples and stressed samples (* *p* ≤ 0.5; ** *p* ≤ 0.05; *** *p* ≤ 0.005). G1–16: different expression groups.

Based on RNAseq results obtained from the independent analysis of leaf and root tissues from strawberry (*Fragaria* × *ananassa*) plants subjected to drought (PEG treatment) and salinity (NaCl-induced salt stress), thirty-five *NAC* genes (*FaNAC*) were identified that exhibited differential expression (fold change ≥ ±2; *p*-value ≤ 0.05; FPKM ≥ 5) under the stress conditions analyzed. These genes were subsequently classified into sixteen distinct expression groups (groups 1–16), based on their expression patterns determined by RT-qPCR (Figure 1 and Figure 2; Supplementary Table S1). The expression profiles of the identified *FaNAC* genes did not always coincide between the two methodologies, probably due to the greater specificity and sensitivity of the RT-qPCR technique vs. RNAseq (Supplementary Table S1). The genes contained within groups 1–9 invariably exhibited upregulation of expression, under both drought and salinity conditions. Pursuant to these expression profiles, the genes were grouped as follows: Group 1 (*FaNAC3*, *FaNAC5*, *FaNAC6*, *FaNAC8*, *FaNAC10*, *FaNAC12*, *FaNAC16*, *FaNAC20*, *FaNAC29* and *FaNAC92*), exhibits higher expression in both leaves and roots under drought and salinity conditions; Group 2 (*FaNAC2*, *FaNAC14* and *FaNAC17*), whose expression is up-regulated in leaves under both drought and salinity conditions, whereas in the roots it is induced only under salinity conditions; Group 3 (*FaNAC22* and *FaNAC26*), whose expression is induced in both leaves and roots under salinity conditions, while in roots it is induced only under drought conditions; Group 4 (*FaNAC21*), whose expression is induced in all tissues and under all stress conditions analyzed, except in leaves subjected to salt stress; Group 5 (*FaNAC9*) comprises the only *FaNAC* gene whose induction has been observed in leaves and roots under drought stress conditions, but only in leaves under salt stress; Group 6 (*FaNAC4* and *FaNAC13*), whose expression is up-regulated in leaves under drought conditions and in roots under salt stress; Group 7 (*FaNAC24*), showed up-regulation exclusively under salinity conditions; Group 8 (*FaNAC15*) increased its expression in the leaves only under drought conditions; and Group 9 (*FaNAC28*) showed higher expression exclusively in the roots under salinity conditions. In addition, the genes contained within groups 10–14 manifested divergent expression profiles, depending on the specific tissue or stress condition under study. The following categorization was used: Group 10 (*FaNAC25*) is characterized by induced expression in both leaves and roots under salinity conditions and by repressed expression in leaves under drought conditions; Group 11 (*FaNAC18*) is distinguished by its induction in leaves and repression in roots under drought conditions, exhibiting behavior opposite to that described for salinity conditions; Group 12 (*FaNAC38* and *FaNAC40*) is distinguished by the up-regulation of gene expression in leaves under drought conditions and down-regulation in leaves and roots under salinity conditions; Group 13 (*FaNAC31*) exhibited induction in the roots under drought conditions and repression in the leaves under saline conditions; and Group 14 (*FaNAC27*), which includes the only gene showing expression specificity in response to salinity, with its expression being induced in the roots and repressed in the leaves under such stress. Finally, these results also showed that the genes contained in groups 15 (*FaNAC35*) and 16 (*FaNAC33*) invariably exhibited repressed expression in the corresponding tissues (Supplementary Table S1). Thus, *FaNAC35* exhibits marked repression in both leaves and roots under both types of abiotic stresses, whereas *FaNAC33* undergoes specific downregulation in the leaves under both drought and salinity conditions. Therefore, although most genes exhibit a discernible induction profile in response to drought or salinity stress in one or both tissues examined, a notable exception is constituted by *FaNAC25*, *FaNAC18*, *FaNAC38*, *FaNAC40* and *FaNAC31,* which respond to both abiotic stresses in an opposing manner. In contrast, some of the analyzed *FaNAC* genes showed response specificity to a specific abiotic stress. This is the case of *FaNAC23*, *FaNAC24*, *FaNAC28* and *FaNAC27* which have been shown to respond specifically to salt stress, and for *FaNAC11* and *FaNAC15*, whose response to water stress has been demonstrated. Similarly, *FaNAC11* and *FaNAC15*, as well as *FaNAC28* and *FaNAC23*, exhibit tissue expression specificity, as they are induced exclusively in leaves and roots, respectively. The former are induced under drought conditions and the latter under salinity conditions.

The results obtained therefore suggest that the *FaNAC* genes identified play an important role in the strawberry plant’s response to the abiotic stresses analyzed, as they repress and/or induce their expression in response to these stresses. This behavior has previously been described in many *NAC* genes of higher plants in response to drought and salinity. Thus, it has been documented that *ONAC066* is expressed in rice seedlings when subjected to these abiotic stresses [52]. Likewise, higher expression of *RsNAC023*, *RsNAC080* and *RsNAC145* have been observed in radish leaves and roots under comparable stress conditions [53]. Furthermore, some *NAC* genes also exhibit a specific expression pattern in response to a particular stress (drought or salinity) or in a specific tissue (leaf or root). This is exemplified by *FaNAC11* and *FaNAC15*, which exhibit specific expression profiles in response to water stress, as does *TaNAC6-3B*, whose overexpression in wheat has been shown to improve drought tolerance in these plants by inducing the *NCED* and *TaLEA1-2B* genes [54]. Alternatively, *FaNAC23* and *FaNAC28* have been observed to be expressed exclusively in the roots under conditions of high salinity, just as occurs with *GmNAC19* and *GmGRAB1* [55]. Analogous outcomes have been witnessed for *SlNAC35*, which is expressed in tomato roots. Overexpression of *SlNAC35* in transgenic tobacco plants has been shown to enhance abiotic stress tolerance while stimulating root growth and increasing the number of lateral roots [56]. In the case of *TaRNAC1*, which is predominantly expressed in roots, its overexpression in wheat resulted in increased root length, biomass and drought tolerance and improved grain yield under water limitation [57].

Furthermore, RT-qPCR analysis revealed the downregulation of *FaNAC35* under drought and salinity conditions in all tissues analyzed; of *FaNAC27*, *FaNAC31*, *FaNAC38* and *FaNAC40* under salinity conditions; and of *FaNAC18* and *FaNAC33* in the presence of both types of stress. Similarly, *FaNAC25* showed reduced expression in response to drought conditions. These findings suggest that these *FaNAC* genes function as negative regulators of the strawberry plant’s ability to resist the stresses examined. This behavior has previously been described for other genes, including *ZmNAC55* and *OsNAC092*. Thus, transgenic maize lines with reduced *ZmNAC55* expression exhibited enhanced drought tolerance [58]. Similarly, *OsNAC092* knockout transgenic rice lines exhibited an enhanced antioxidant system and an improved GSH/GSSG ratio, which accelerated ROS scavenging to counteract oxidative damage and improved plant survival under water deficit conditions [59]. Also, *GmNAC2* is a negative modulator of flavonoid biosynthesis in soybean, compounds that promote salt tolerance in this plant [60]; and *MsNAC2a* is a negative modulator in alfalfa, where resistance to salt stress is induced through the interaction of MsNAC2a, both in vitro and in vivo, with the AP2/EREBP-type transcription factor MsEREBP1 [61]. Consequently, our results seem to indicate that among the *FaNAC* genes involved in the strawberry plant’s response to drought and salinity, there is a subgroup of genes that responds generally to both abiotic stresses, whereas another subgroup exhibits tissue and/or stress-specific expression. Furthermore, it has been observed that some of them improve drought or salinity tolerance through positive regulation way, while others achieve the same objective via negative regulation. This highlights the existence of diverse mechanisms by which the identified *FaNAC* genes regulate drought and/or salinity tolerance in the strawberry plant. The observation that a subset of *FaNAC* genes undergoes marked up-regulation under both stress conditions suggests that these genes may play fundamental regulatory roles in the response to such environmental stresses and that the modulation of their expression is likely to facilitate the strawberry plant’s adaptation to these adverse conditions. This finding is consistent with the behavior described for NAC transcription factors in other plant species, in which their overexpression enhances drought and/or salinity tolerance. For instance, in pear (*Pyrus betulifolia,* Bunge), the overexpression of PbNAC155 significantly induces salt stress tolerance through its interaction with PbABL5, a key transcription factor in ABA signaling [62]. Overexpression of *LbNAC55* in *Limonium bicolor* also significantly increased the salinity tolerance of this species [63], as did that of *ONAC005*, a gene induced by NaCl and ABA whose overexpression enhanced salinity tolerance in rice by reducing Na^+^ ion accumulation and inducing the expression of trehalose-6-phosphate synthase 8 (OsTPS8), which in turn stimulates the formation of a hydrophobic barrier in the root endodermis through suberin deposition [64]. Similarly, overexpression of *NtNAC236* in tobacco plants enhanced their drought tolerance, as evidenced by transcriptomic analysis revealing that *NtNAC236* regulates 458 target genes that are differentially expressed under water stress [65]. Furthermore, expression analyses confirmed tissue-specific expression or expression in response to specific stresses for some of the *FaNAC* identified [55,66].

### 2.2. Phylogenetic Studies of the Selected FaNAC Transcription Factors

**Figure 3:**
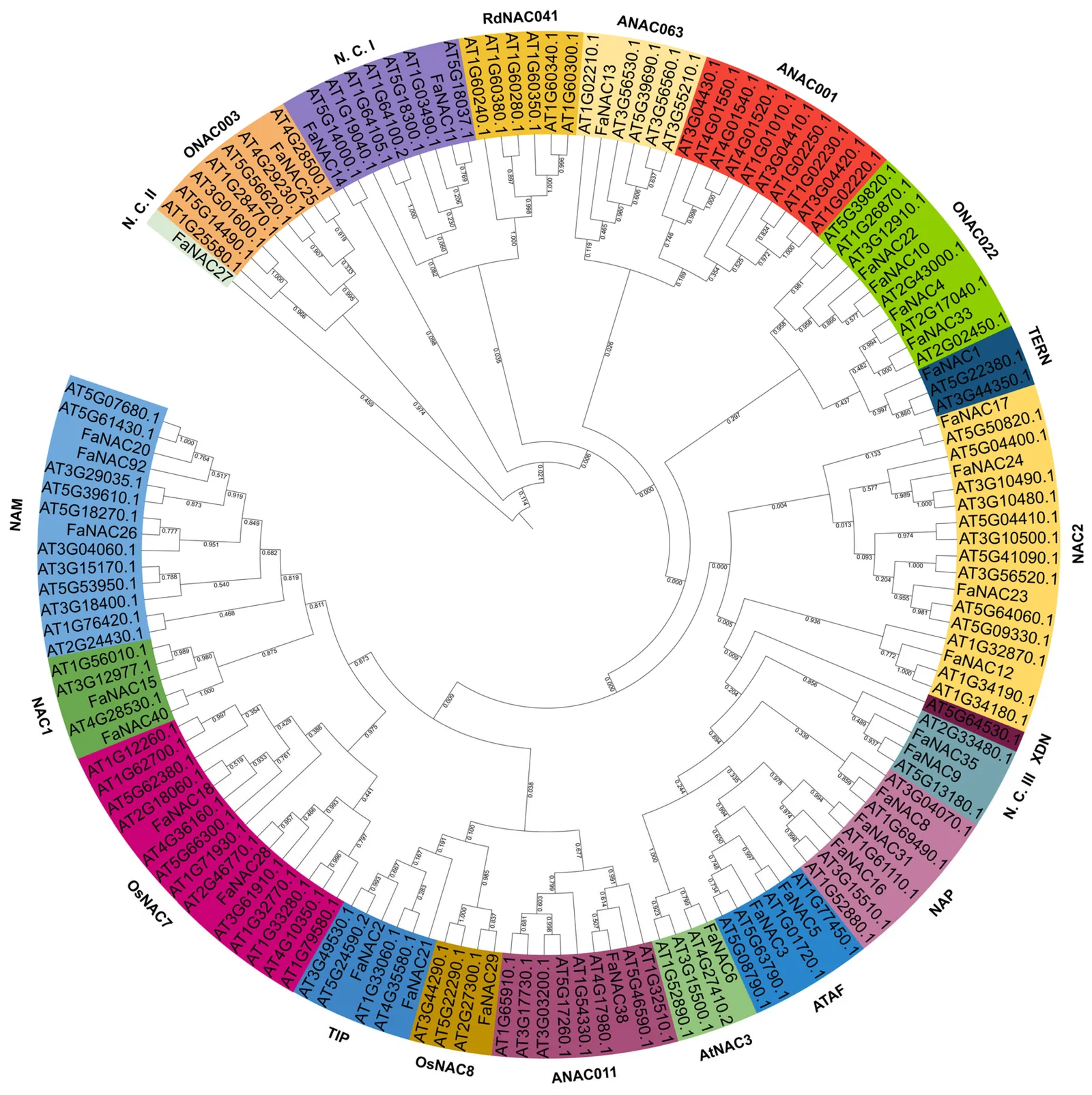
Phylogenetic tree of *Arabidopsis thaliana* NAC (AtNAC) and strawberry (*Fragaria* × *ananassa*) FaNAC proteins show differential expression under drought and salinity conditions.

To ascertain the potential evolutionary trajectory of the selected FaNAC TFs in response to drought and salinity, a comparative phylogenetic tree was constructed with the AtNAC proteins identified in *Arabidopsis* (Figure 3). The FaNAC and AtNAC proteins were grouped into 20 subclades. However, a few FaNAC proteins did not cluster with any recognized AtNAC clade and were therefore assigned to three novel groups, designated New Class I–III. Of the identified subclades, the NAC2, OsNAC7 and NAM subclades contained the largest number of members, with 17, 15 and 14 TFs respectively. This finding suggests the potential for functional conservation of these genes within *F.* × *ananassa*. The data demonstrates that subclades ANAC001 and ONAC022 both included 10 members, the NCI and ANAC011 included 9 members, and subclade NAP and ONAC003 included 8. Other subclades, such as ANAC063, ATAF and TIP included 6 members, while the remaining smaller subclades, such as NAC1, NCII, AtNAC3, OsNAC8, NCIII, TERN and XDN included between 5 and 1 members. Of particular interest is the observation that the ANAC001, RdNAC041 and XDN subclades did not include any of the FaNAC proteins related to drought and salinity response, suggesting that this lineage has diverged in *F.* × *ananassa*. Moreover, the NCII subclade’s composition, exclusively comprising one FaNAC TF absent in *Arabidopsis*, may imply a distinct evolutionary trajectory of this gene across species. This suggests a targeted adaptation of the FaNAC proteins to the abiotic stresses examined in *F.* × *ananassa*. Furthermore, the emergence of the NCI-III subclades in strawberry may be associated with an expansion of NAC proteins in this plant, suggesting that this family of TFs has diversified in this species due to specific evolutionary pressures. Furthermore, the NCII subfamily includes FaNAC27, which exhibits no sequence similarity with any AtNAC protein, thereby indicating the potential for this TF to fulfil specific functions in strawberry. It was observed that the NAC proteins associated with the response to drought and salinity in strawberries are distributed evenly across all the identified subfamilies, apart from the ANAC001, RdNAC041 and XDN subfamilies. This absence in *F.* × *ananassa* indicates that these proteins may have been lost in this plant during its evolution, thus highlighting the divergent evolutionary trajectories of *Arabidopsis* and strawberry.

Some of the identified FaNAC proteins were found to be members of the ATAF, NAP, and OsNAC003 subfamilies, which have been demonstrated to be involved in stress response processes [67,68,69], and in the ONAC022 subfamily, which includes the transcription factor JUB1, whose overexpression significantly increases drought stress tolerance in *Arabidopsis* [70]. The NAC2 subfamily includes ANAC013, which has been demonstrated to participate in the stress response in *Arabidopsis thaliana* [71]. Specifically, ANAC013 acts on the mitochondrial retrograde regulatory mechanism (MRR) that occurs in the plant during stress, when mitochondria send signals to the nucleus to direct the expression of genes that are induced in response to these conditions [71]. In addition, the FaNAC6 protein is in the AtNAC3 subfamily, which also includes RD26/ANAC072. This protein has been extensively documented in scientific literature, where it is widely acknowledged for its role in the activation of genes involved in the ABA biosynthesis pathway in response to abiotic stress [72]. Furthermore, the orthologous genes of *FaNAC6* have been shown to possess analogous functions in response to stress in various plant species, including *Camellia sinensis*, *Madhuca longifolia*, and *Apium graveolens* [66,73,74]. The results obtained appear to indicate that the functions of the identified FaNAC proteins are analogous to those described in the literature for other NAC proteins in response to abiotic stresses.

### 2.3. Chromosomal Localization and Synteny Analysis of FaNACs with Variable Expression Under Drought and Salinity Conditions

**Figure 4:**
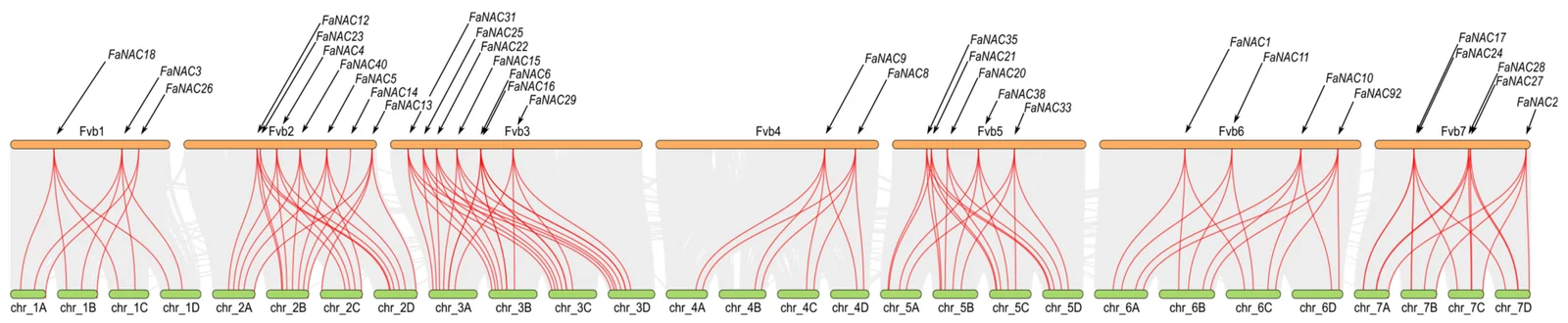
Analysis of interspecific synteny between *Fragaria vesca* and *Fragaria* × *ananassa* and chromosome mapping of *FaNAC* genes with differential expression under drought and salinity stress. The grey lines in the background represent syntenic blocks. The red lines highlight the syntenic *NAC* gene pairs.

To evaluate the evolutionary patterns exhibited by the *FaNAC* genes identified under the stress conditions analysed, a collinearity analysis was performed between *Fragaria vesca* and *Fragaria* × *ananassa*. The chromosomal location of the genes in question was determined in both the *F. vesca* and *F.* × *ananassa* genomes (Figure 4). The results demonstrated that the *NAC* genes responsive to drought and salinity were distributed across the seven chromosomes of *F. vesca* and the 28 chromosomes of *F.* × *ananassa*, with chromosomes 2, 3 and 5 of both species exhibiting a higher number of *NAC* genes. In general, the majority of the analyzed genes present in *F. vesca*, regardless of stress, had four homeologous copies in the octoploid specie *F.* × *ananassa*. Each copy corresponds to one of the four subgenomes (A, B, C and D) contained in this species due to its polyploid nature. The results indicate a high degree of chromosomal conservation of the analyzed genes between both species, which supports the hypothesis that gene duplication is a key factor in the expansion of the *NAC* family in *Fragaria* and in its functional innovation. However, several clear exceptions to this behavior were observed. Specifically, there is only one copy of *FaNAC14* on Chr_2B of *F.* × *ananassa*, two copies of *FaNAC26* (Chr_1A and 1B) and *FaNAC23* (Chr_2B and Chr_2D), and three copies of *FaNAC1* and *FaNAC11* (Chr_6A, 6B and 6C), respectively. The results indicate that, although most *NAC* genes are conserved in the four subgenomes of cultivated strawberry, there are events of loss that could affect the function and regulation of these *NAC* genes in *F.* × *ananassa*. In addition, this finding suggests that this species may exhibit greater resilience to environmental stress due to functional redundancy. This hypothesis, together with the existence of *FaNAC* genes that can induce or repress their expression in response to stress, suggests high genomic plasticity in strawberries that would allow the plant to develop a more effective evolutionary strategy against different abiotic stresses. 

### 2.4. Analysis of Conserved Motif and Domains

**Figure 5:**
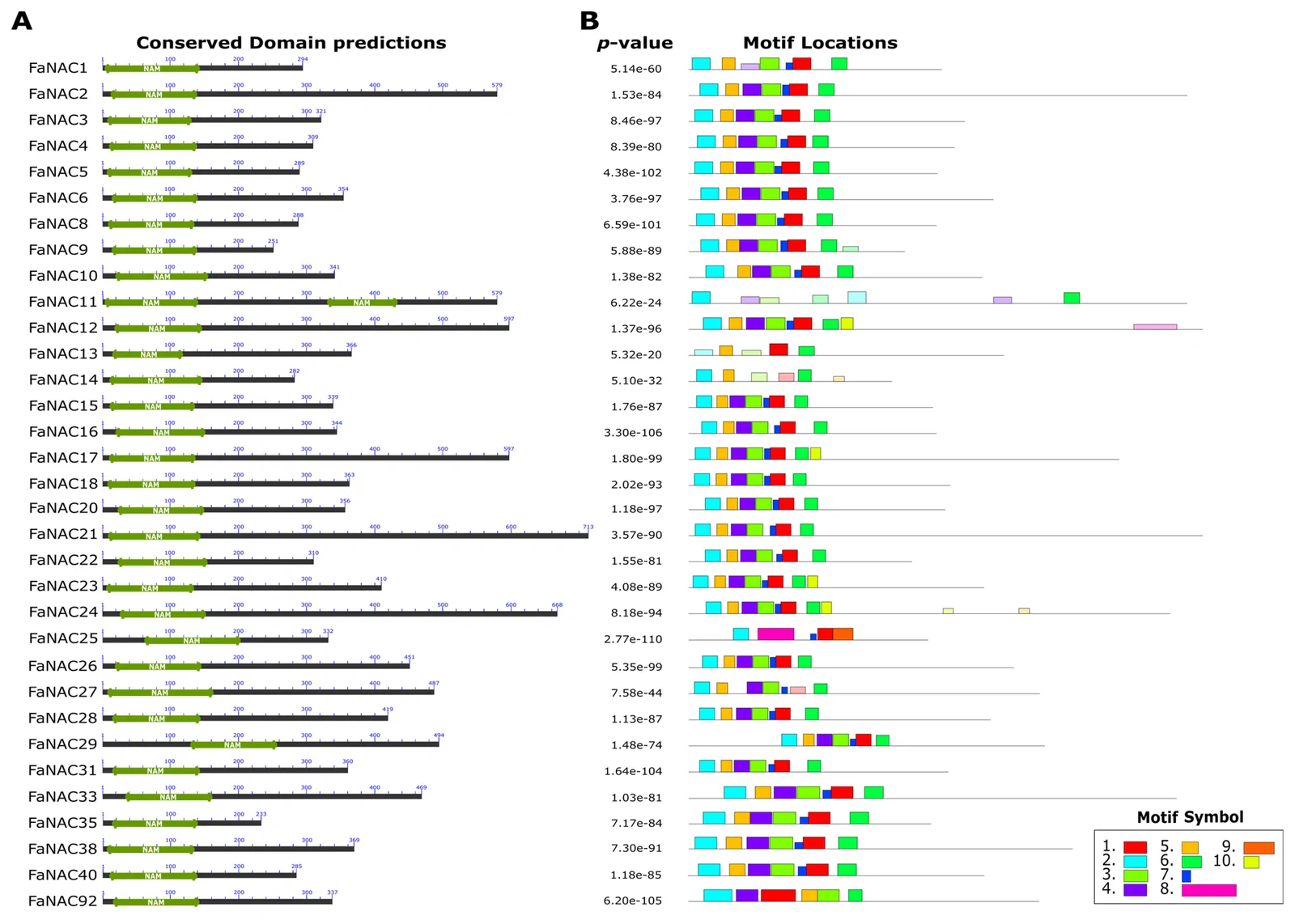
Conserved NAC domains (**A**) and conserved motifs (**B**) identified in FaNAC proteins with differential expression during the strawberry plant response to drought and/or salinity. Ten motifs are shown as boxes in different colors.

An analysis of conserved domains of FaNAC proteins encoded by genes with differential expression under drought and/or salinity conditions was performed (Figure 5A). The study revealed that 97% of these FaNAC proteins contained a single conserved NAM domain, a characteristic feature that is hallmark of these proteins [75,76]. This domain is critical for the transcriptional regulation and functional diversity of NAC proteins. In the majority of cases, the NAC domain was found to be concentrated at the N-terminal end (between amino acids 1–160), although in two cases (FaNAC25 and FaNAC29) the NAC domain was located somewhat further from the N-terminal end (between amino acids 60–200 and 120–260, respectively) (Supplementary Table S2). Nevertheless, the data show a high degree of conservation of this domain and suggest similar biological functions for all these proteins. Furthermore, the predominant conservation of the NAC domain distribution pattern indicates that it is key to the function of *NAC* genes in plants (Figure 5A). This phenomenon has been observed in the NAC genes of the sunflower, where it has been found that the conserved NAC domain at the N-terminal end of these transcription factors maintains their ability to bind to DNA [77]. Notably, the protein FaNAC11 exhibited two NAC domains. This phenomenon could be ascribed to a gene duplication event, which, in turn, has the potential to enhance the binding affinity of these TFs to DNA and augment the complexity of their interactions [76]. In general, the analysis of the FaNAC proteins associated with the response of the strawberry plant to drought and/or salinity revealed the presence of at least one NAC domain, highlighting the importance of this domain in the response to abiotic stress.

Concurrently, analysis with the MEME online tool identified 10 conserved motifs in FaNAC proteins (Figure 5B). Motifs 1–7 exhibited a high degree of conservation and were present in a significant proportion of FaNAC proteins. Motifs 1–7 were present in 87.8–100% of the proteins analyzed. The consensus sequences of the identified motifs are provided in Supplementary Figure S1. Additionally, comparison of these sequences with the conserved A–E subdomains of the NAC domain described by Ooka et al. [78] allowed the putative association of Motifs 1–7 and 9 with specific NAC subdomains (Supplementary Figure S1). Specifically, Motif 2 was associated with subdomain A, Motif 5 with subdomain B, Motifs 3 and 4 with subdomain C, Motifs 7 and 1 with subdomain D, and Motifs 6 and 9 with subdomain E. These results show that several of the conserved MEME motifs are located within specific regions of the NAC domain. In contrast, Motifs 8–10 were detected in a low proportion of the FaNAC proteins analysed, and Motifs 8 and 10 could not be assigned to the conserved A–E NAC subdomains.

### 2.5. Prediction of Subcellular Localization of FaNAC Proteins

The distribution of FaNAC proteins was analyzed in silico and the results obtained predicted that the majority were localized in the nucleus (93.93%), while FaNAC17 and FaNAC23 were found in the cytoplasm and FaNAC11 in the plastid (Supplementary Table S3). The predominance of FaNAC genes within the cell nucleus is consistent with the function of a transcription factor. However, the fact that FaNAC11 is localized in the plastid, and moreover, is the only FaNAC protein containing two NAC domains (Figure 5A), could suggest a specific function for this protein within that organelle. Furthermore, the prediction of the subcellular localization of NAC proteins outside the nucleus has previously been described in other plants such as carnations [6] and in citrus [79]. This distinct localization pattern could indicate new functions for FaNAC17 and FaNAC23 proteins through the mediation of inter-organelle signaling and membrane trafficking [80], or a pre-stress extranuclear localization of these proteins. The latter has been described for ONAC023, which is localized in the cytoplasm and translocated to the nucleus during drought or heat stress in rice [31]. However, these predictions are theoretical, and experimental validation will be required to confirm the actual subcellular localization of these proteins.

### 2.6. Analysis of Cis-Acting Elements in the FaNAC Promoters

**Figure 6:**
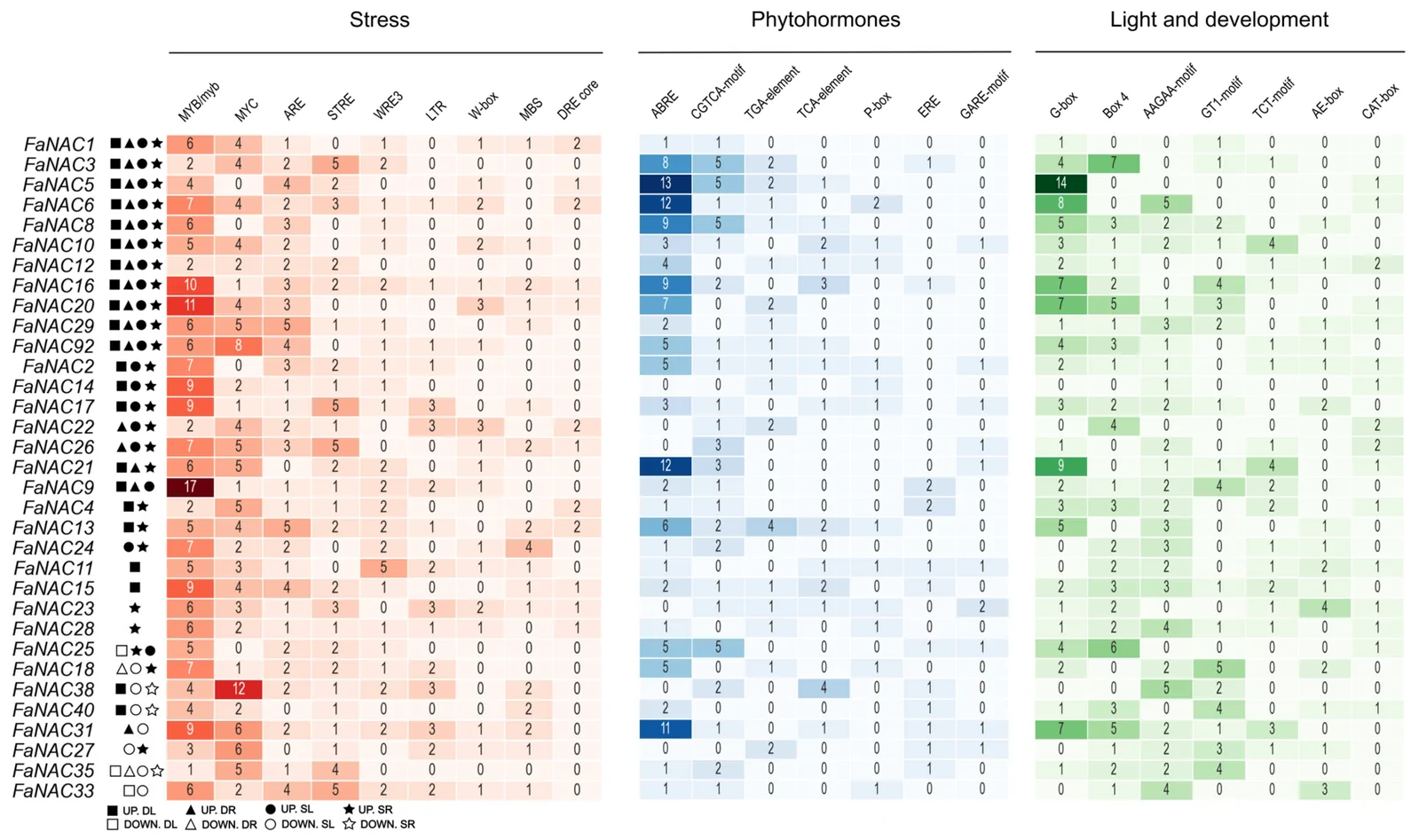
Heatmap of the main cis-regulatory elements identified in the promoter sequences of *FaNAC* genes with differential expression under drought and/or salinity. The cis-elements were classified into three functional categories: stress-sensitive elements (red), phytohormone response elements (blue), and light and development-response elements (green). Element quantity differences are displayed through a color-scale gradient. DL: drought in leaves; DR: drought in roots; SL: salinity in leaves; SR: salinity in roots.

Since cis-regulatory sequences in the promoter region of a gene are essential for its functionality [81], the PlantCARE web server was used to analyze the 2 kb promoter region of the *FaNAC* genes with differential expression under drought and salinity conditions. Of the total cis-regulatory elements identified in this analysis, the most representative were selected and classified into three functional categories (Figure 6). The first group comprised stress-sensitive elements, including recognition elements of TFs, MYBs and MYCs (all related to the abiotic stress response), as well as AREs (anaerobic induction), STREs (stress response), WRE3 (wound-responsive elements), LTRs (low temperature response), W-boxes (drought-related WRKY transcription factor binding sites), MBSs (drought-related MYB transcription factor binding sites) and DRE-cores (drought stress). The second group comprised phytohormone response elements such as ABRE (abscisic acid response), TGA-element (auxin response), CGTCA-motif (MeJA response), TCA-element (salicylic acid response), ERE (ethylene-responsive element), P-box and GARE-motif (both related to gibberellin response). The final group included light-response elements, such as the TCT-motif, GT1-motif, G-box, Box4, and AE-box, as well as development-related elements, such as the AAGAA-motif (which regulates endosperm expression) and the CAT-box (which is meristem-specific). Analysis revealed that the analyzed promoters were enriched in abiotic stress response elements, followed by hormone response elements. These findings suggest that these genes play a role in stress response through different signaling pathways. Furthermore, the abundance of cis-elements recognizing MYB/MYC TFs, whose involvement in abiotic stress response processes has been widely demonstrated [82,83,84], supports the idea of a key role for these *FaNAC* genes in strawberry adaptation to drought/salinity stress. The promoters analyzed also exhibited a high number of elements associated with the response to ABA and MeJA. Both hormones play a pivotal role in plants in response to various abiotic stresses, including drought and salinity. Under drought conditions, ABA accumulates in guard cells, thereby activating ion channels and inducing stomatal closure to minimize water loss. Concurrently, the expression of stress response genes is stimulated and modulated by ABA, thereby enhancing the synthesis of protective proteins and metabolites [6,85]. In a similar manner, under saline conditions, ABA binds to PYR/PYL/RCAR receptor proteins, forming a complex that stimulates the activity of SnRK2s which, in turn, activate ABF transcription factors that specifically bind to ABRE elements present in the promoters of numerous genes related to stress response [4]. In the case of MeJA, at the molecular level, this hormone participates in the activation of antioxidant systems, the accumulation of amino acids and soluble sugars, and the induction of JA-dependent gene expression, including JAZ, AOS1, AOC, and LOX2 genes, and to interact with other phytohormones such as ABA, or with TFs from the MYC or bHLH families [7]. 

Specifically, in rice, it has been demonstrated that drought upregulates OsbHLH148; this TF in turn induces the expression of OsDREB1 and OsJAZ, which are related to the response to drought stress and the JA signaling pathway, respectively [86]. In a similar manner, an increase in the concentration of endogenous JA has been demonstrated to enhance the plant’s tolerance to salinity by maintaining the homeostasis of ROS in tomato [87]. It has also been reported that, under drought conditions, exogenous application of JA in strawberries increased the activity of antioxidant enzymes, including malondialdehyde (MDA), H_2_O_2_ and proline content [88]. Similar behavior was observed in maize, where the application of MeJA has been shown to reduce the harmful effects of drought-induced oxidative stress by decreasing MDA levels, LOX activity and H_2_O_2_ concentration, whilst simultaneously increasing proline content, total soluble carbohydrates and sugars, and antioxidant enzyme activities (CAT, POD and SOD). This is due to the levels of ABA and MeJA present in stressed leaves and roots [89]. Furthermore, studies have demonstrated the interaction between JA and ABA signal transduction pathways. For instance, the transcription factor MYC2 modulates the AOC1 gene, which is pivotal in JA biosynthesis [90], whilst concurrently contributing to the ABA-mediated drought stress response [91]. Similarly, OsbHLH148 rapidly increases its transcription levels in response to treatment with MeJA or ABA, and the TF ORA47 acts as a target gene in *Arabidopsis* plants subjected to water stress in both ABA and JA biosynthesis [92]. Consequently, both hormones appear to be essential in the response of plants to drought and salinity stress. The presence of numerous cis-elements regulated by both phytohormones in the promoters of the *FaNAC* genes analyzed suggests that these genes may play a central role in the response to drought and salinity stress in strawberries and that their expression may be influenced by the levels of ABA and MeJA present in stressed leaves and roots. However, this promoter analysis is based on bioinformatic prediction and the presence of cis-elements associated with the response to ABA and MeJA only indicates a potential regulatory capacity of these phytohormones on the analyzed promoters; it does not demonstrate that the identified cis-elements are functional or that these phytohormones directly regulate each promoter. Functional validation of these putative cis-regulatory elements by promoter-reporter assays (e.g., dual-luciferase assays) will be required in future studies.

### 2.7. Expression Profiling of FaNAC6

**Figure 7:**
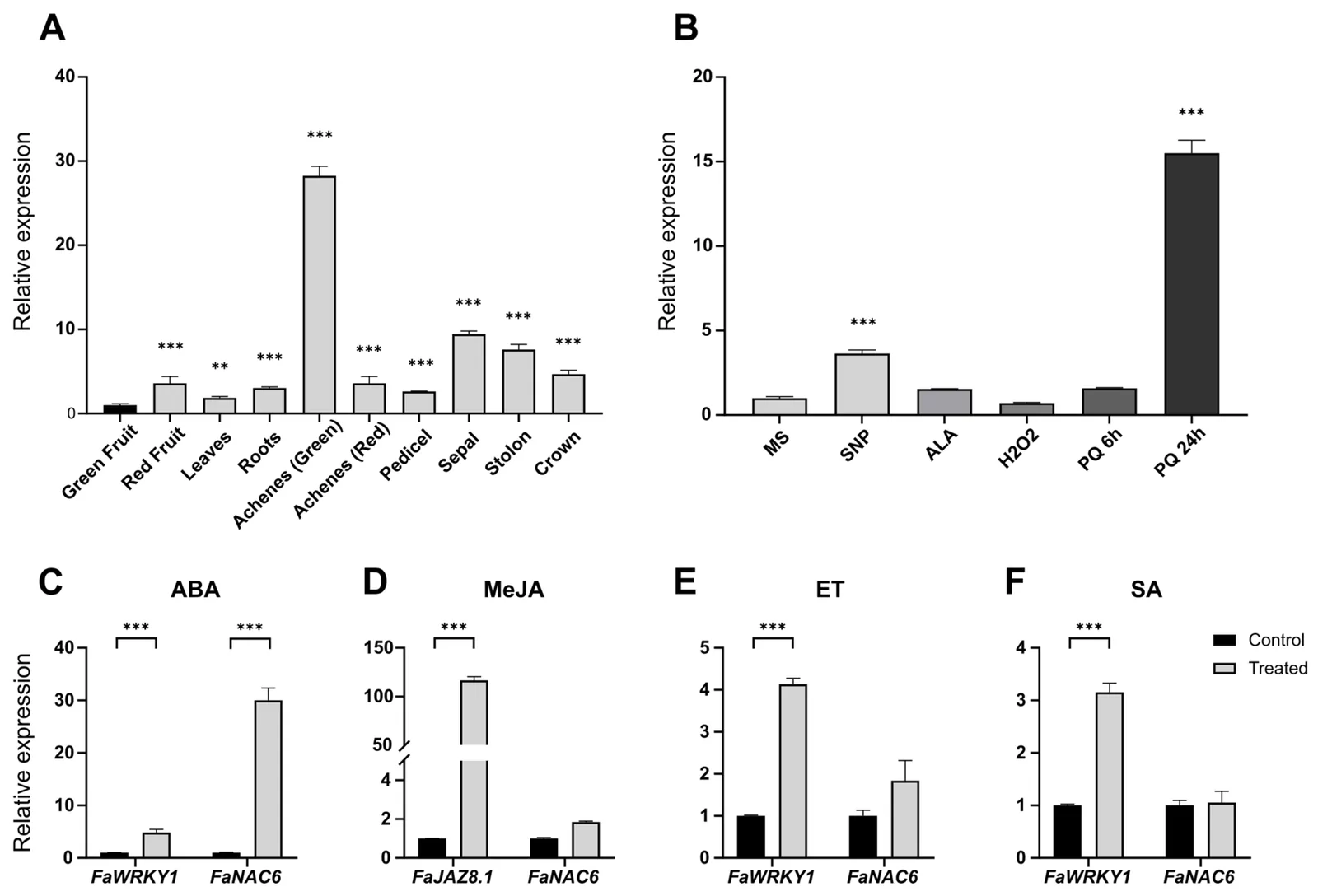
*FaNAC6* expression profile determined by RT-qPCR in response to oxidative stress and various hormonal treatments. (**A**) Expression profile of *FaNAC6* in fruit receptacles at different developmental stages and in vegetative tissues of *Fragaria* × *ananassa* cv. Chandler. (**B**) Relative expression of *FaNAC6* in leaves following treatment with stress-related compounds: 72 h with ALA and SNP; 5 h with H_2_O_2_; 6 and 24 h with paraquat (PQ). (**C**–**F**) Relative expression levels of hormone-responsive marker *FaWRKY1* [93] and *FaJAZ8.1* [94] genes and *FaNAC6* gene in leaf tissue following a 6 h treatments with different phytohormones: (**C**) ABA 0.1 mM; (**D**) MeJA 0.1 mM; (**E**) ET 100 mg L^−1^; and (**F**) SA 5 mM. Transcript levels were quantified by RT–qPCR using gene-specific primers and normalized against the *GAPDH2* gene. Expression values were calculated relative to receptacle stage G1, corresponding to the green developmental stage with lowest *FaNAC6* expression (**A**), or to untreated controls maintained in MS medium (**B**–**F**), which were set to 1. Data represent mean ± SEM of five independent biological replicates. Statistical significance was assessed using a two-tailed Student’s *t*-test; ** *p* < 0.05, *** *p* < 0.005.

Among all the *FaNAC* genes identified that showed differential expression in response to drought and salinity, we selected *FaNAC6* for further functional characterization, as it exhibited high levels of expression in response to both abiotic stresses. Using RT-qPCR, we conducted a comprehensive analysis of its transcriptional expression levels in diverse vegetative tissues, encompassing leaf, root, green and red achenes, pedicel, sepal, stolon and crown, as well as fruit tissue, including green and red (Figure 7A). The analysis revealed *FaNAC6* expression in all tissues examined, with the highest levels of expression observed in sepals and stolons, and notably in green achenes, where its expression was found to be 28 times higher than that detected in the green fruit. Consequently, *FaNAC6* does not exhibit tissue-specific expression (Figure 7A). Furthermore, the high expression levels observed in green achenes could be attributed to the significant degree of dehydration this tissue undergoes, given its classification as a seed. This finding provides a potential explanation for the induction of genes associated with the response to water deficit, such as *FaNAC6*.

A similar study was conducted on the expression of *FaNAC6* in plants treated with ALA, SNP, H_2_O_2_ and PQ (Figure 7B). ALA has been shown to improve salinity tolerance in strawberry plants through a process involving nitric oxide (NO) and hydrogen peroxide (H_2_O_2_) [95]. Indeed, NO has been found to play a role in plant resistance to various abiotic stresses [96]. Consequently, the application of sodium nitroprusside (SNP), an NO donor, to strawberry plants could simulate a stress situation, simulating and triggering an appropriate stress response. Similarly, it has been observed that H_2_O_2_ enhances salt tolerance by inducing NADPH oxidases in both *Arabidopsis* [97,98] and tobacco [99]. Conversely, PQ has been shown to induce oxidative stress through the generation of superoxide radicals, thereby simulating a stress scenario for the plant. Treatments with ALA and H_2_O_2_ and short-term treatment with PQ did not affect *FaNAC6* gene expression in the leaves. However, it was found that treatments with PQ and SNP significantly induced *FaNAC6* expression in this tissue after 24 and 72 h, respectively (Figure 7B). These results suggest that NO and superoxide radicals can act as signaling molecules to regulate *FaNAC6* expression during stress processes. This phenomenon has been documented in oats, where an in vivo increase in NO has been observed under moderate or high-water stress conditions in the susceptible cv. Flega [100]. Furthermore, exogenous application of SNP has been shown to reduce lipid peroxidation and stimulate antioxidant systems in *Origanum majorana* and *Scrophularia striata* under drought stress [101]; meanwhile, under salt stress conditions, SNP significantly increased the activity of antioxidant enzymes (SOD, CAT, APX, POX, and polyphenol peroxidase) in *Panax ginseng* and *Oryza sativa* L. protecting them from such stress [102]. Also, in *Brassica napus*, it has been demonstrated that treatment with NO exerts a regulatory effect on the NaCl-induced toxicity, simultaneously increasing phenolic content and PAL enzyme activity in plants subjected to this stress [103]. Furthermore, it has been reported that the synthesis of molecules such as superoxide anion (O_2_^•−^) and hydroxyl radicals (^•^OH) can stimulate the response to abiotic stress in plants. For instance, in rice, it has been shown that high salinity conditions induce antioxidant enzymes and genes associated with the anthocyanin biosynthetic pathway [104]. Consequently, the observed behavior of the *FaNAC6* gene in plants treated with SNP and PQ is consistent with what has been previously described in the literature for other higher plants. 

The expression of *FaNAC6* was also quantified by RT-qPCR in leaves of *Fragaria* × *ananassa* cv. Chandler treated individually with ABA, MeJA, ethephon (ET) and salicylic acid (SA). It has been shown that these phytohormones stimulate transcriptional cascades that activate families of TFs, including NACs, which regulate the expression of genes responding to water and salt stress [105,106]. Thus, the role of ABA in plants is of great importance under drought conditions, as it induces stomatal closure, a vital process for preventing water loss. Furthermore, it triggers a long-distance systemic response through transport from the leaves to the rest of the plant tissues [106]. Subsequently, ABA binds to its receptors (PYR/PYL/RCARs), thereby activating the canonical ABA signaling pathway [106]. Furthermore, jasmonates frequently act synergistically with ABA to enhance osmotic adjustment and activate drought-defense mechanisms [107]. Specifically, under drought conditions, they modulate stomatal closure, the detoxification of ROS, and the expression of genes inducible by this type of stress. Similarly, ET plays a pivotal role in the plant’s response to biotic and abiotic stresses such as pathogen attack, flooding, or high salinity [108]. In rice, the induction of ethylene biosynthesis by *OsARD* overexpression has been shown to enhance the plants’ tolerance to submergence [109]. Nevertheless, the role of ET in the plant’s response to drought remains ambiguous. Our results showed a significant induction of *FaNAC6* expression six hours after exogenous application of ABA, whereas its expression was not significant following treatment with MeJA or ET (Figure 7C–E). The findings of this study suggest a pivotal regulatory role for ABA regarding the *FaNAC6* gene in the strawberry response to drought. This conclusion is consistent with previous research on other plants, including rice [110], tomato [111], and *Arabidopsis* [112]. In contrast, MeJA and ET seem to play a secondary role in this response, despite the documented interaction of both hormones with ABA in response to both water and salt stress [108].

SA has been shown to participate in the drought response, either cooperatively or antagonistically with ABA, depending on the duration and intensity of the stress [108]. It functions as an intracellular signaling molecule, interacting with ROS in specific signal transduction pathways. Also, it has been demonstrated that the exogenous application of SA to *Arabidopsis* and *Brassica nigra* induces the production of hydrogen peroxide (H_2_O_2_) [113]. Indeed, it has been observed that SA, in combination with H_2_O_2_, induces high-temperature tolerance in potatoes, suggesting existence of a shared signaling pathway [114]. During drought, SA contributes to the strengthening of the antioxidant system, the improvement of water retention, the increase in proline content, and the interaction with other phytohormones. Its levels have been shown to increase in species such as barley (*Hordeum vulgare*), olive (*Olea europaea*), and sage (*Salvia officinalis*) under such stress conditions [108]. In our study, the exogenous application of SA to strawberry leaves did not alter the expression of the *FaNAC6* gene (Figure 7F), suggesting that this hormone does not control the expression of this gene in this tissue under the conditions analyzed. This result is consistent with the lack of *FaNAC6* induction observed following treatment with H_2_O_2_, a key intermediate of SA [113], and allows us to suggest that ABA, NO and superoxide radicals are important regulators of *FaNAC6* expression in strawberry. Future studies evaluating different concentrations of the signaling molecules analyzed in this study will provide further insight into the mechanisms regulating *FaNAC6* expression.

### 2.8. Homology of FaNAC6 with Other NAC Genes from Higher Plants Related to Abiotic Stresses

**Figure 8:**
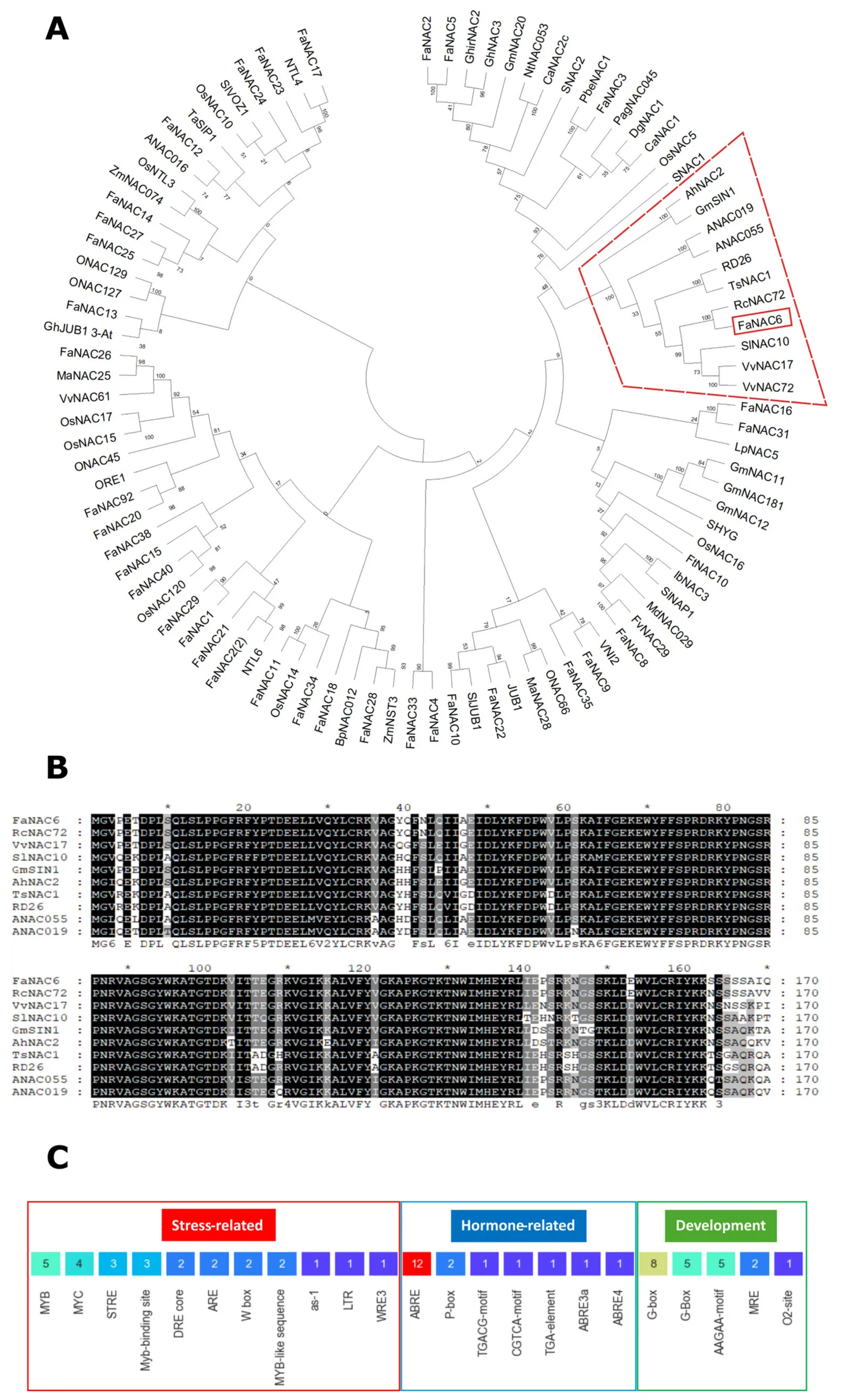
(**A**) Phylogenetic tree of *FaNAC* genes induced by drought and salinity and other *NAC* genes related to the response to these abiotic stresses. The *FaNAC6* gene and its orthologous genes are located within the red dotted line. (**B**) Alignment of the N-terminal end of *FaNAC6* and its orthologous genes *ANAC019*, *ANAC055*, *RD26*, *TsNAC1*, *AhNAC2*, *GmSIN1*, *SlNAC10*, *VvNAC72*, *VvNAC17* and *RcNAC72* (Supplementary Table S4). The boxed regions indicate the conserved NAC domain. Asterisks indicate intermediate positions between numbered residues (**C**) Most significant cis-regulatory elements identified in the *FaNAC6* promoter (2 kb) using the PlantCare software.

Phylogenetic analysis between *FaNAC6* and other *NAC* genes from higher plants revealed their high degree of similarity with *ANAC019*, *ANAC055*, *RD26* (*ANAC072*), *TsNAC1*, *AhNAC2*, *GmSIN1*, *SlNAC10*, *VvNAC17*, *VvNAC72* and *RcNAC72* (Figure 8A). These genes have been implicated in the response to abiotic stress (Supplementary Table S4). *ANAC019* and *ANAC055* are salt tolerance genes, and their overexpression improved the thermotolerance of *Arabidopsis* plants [21,115]. *RD26* has been shown to promote drought tolerance while inhibiting BR signaling in *Arabidopsis* [116]. *TsNAC1*, in collaboration with *TsHD1*, has been demonstrated to significantly enhance the heat and drought resistance of *T. halophila* [117]. *AhNAC2*, in conjunction with *AhAREB1*, serves as a negative feedback regulator of drought-induced ABA biosynthesis in peanut [118]. The overexpression of *GmSIN1* in soybeans promotes root growth and salt tolerance, thus increasing yield under salt stress [119]. Stable overexpression of *VvNAC72* in grapevine and tobacco showed an increase in ROS content, as well as greater resistance to pathogen stress in these plants [120]. *SlNAC10* enhances salt and drought tolerance in *Arabidopsis* transgenic plants, a process that may be attributable to its capacity to regulate proline synthesis [121]. In a similar manner, *VvNAC17* positively regulates drought tolerance in grapes [122] and *RcNAC72* in *Arabidopsis*, where its overexpression increased plant sensitivity to ABA and tolerance to drought stress [123].

Based on the phylogenetic tree, it can be concluded that *FaNAC6* is orthologous to the previously described genes. This suggests that *FaNAC6* could have similar functions in strawberry to those described for them. Furthermore, the alignment of the N-terminal region of *FaNAC6* and its orthologous genes demonstrated the presence of the characteristic conserved NAC domain in all analyzed sequences (Figure 8B). In addition, analysis of its promoter region indicates the presence of a high number of stress- and hormone-inducible cis-regulatory elements (Figure 8C). Indeed, the promoter region of the *FaNAC6* gene is enriched in ABA response ABRE motifs, suggesting that this hormone plays a key role in regulating its expression and supports the induction of its expression observed after the application of ABA to strawberry leaves. However, although *FaNAC6* exhibits high sequence similarity with all the orthologous genes indicated above, phylogenetically it is closer to *RcNAC72* from *Rosa chinensis*, a finding that is logical given that the genera *Rosa* and *Fragaria* belong to the *Rosaceae* family. *RcNAC72* is a transcriptional activator whose expression is significantly induced under conditions of drought, low temperatures, salinity and ABA treatment [123]. Furthermore, its overexpression in *Arabidopsis thaliana* plants has been observed to stimulate their sensitivity to ABA and their tolerance to water stress, as well as inducing the expression of genes related to the response to such stress [123]. Furthermore, the *RcNAC72* promoter contains numerous ABRE elements, which are crucial for its rapid response to exogenous ABA application, DRE core elements, and cis-recognition sequences for MYB- and MYC-type transcription factors, all of which are involved in the response to drought stress [123]. Similarly, the promoter of *FaNAC6* also shows a pattern of cis-regulatory elements analogous to *RcNAC72*, both quantitatively and qualitatively. This finding suggests that the function and regulation of *FaNAC6* expression in strawberry plants may be like that of *RcNAC72* in *Rosa chinensis*. 

### 2.9. Drought and Salinity Tolerance of FaNAC6-Overexpressing Nicotiana benthamiana

To determine the role of *FaNAC6* in the response to drought and salinity, transgenic *Nicotiana benthamiana* plants overexpressing this gene (*FaNAC6*-OX) were generated. In addition, the expression level of *FaNAC6* was analyzed by RT-qPCR in both the generated transgenic lines and the control lines (plants transformed with the empty vector). A total of 29 independent transgenic lines were obtained, and among them, lines L23, L25, and L29 were selected due to their higher levels of *FaNAC6* expression (Supplementary Figure S2). Notably, the selected transgenic lines did not exhibit appreciable phenotypic alterations, despite the effective and robust overexpression of the *FaNAC6* gene.

**Figure 9:**
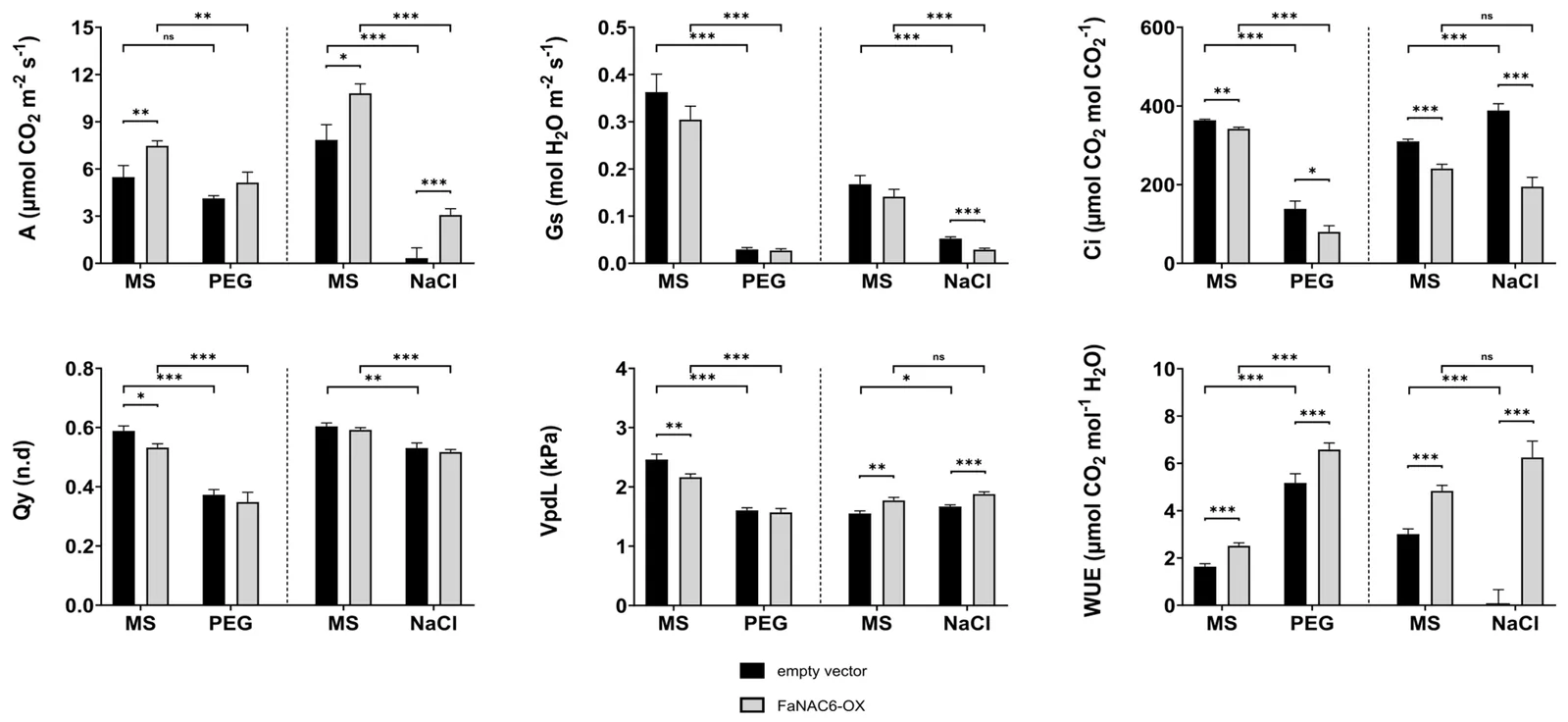
Physiological parameters measured in transgenic *N. benthamiana* plants overexpressing *FaNAC6* gene (*FaNAC6*-OX) and in plants transformed with the empty vector (control). These parameters were measured in unstressed plants and in plants subjected to drought (PEG) or salinity (NaCl). A: net assimilation rate; Gs: stomatal conductance; Ci: intercellular CO_2_ concentration; Qy: instantaneous quantum yield; VpdL: leaf vapour pressure deficit; WUE: water use efficiency. MS: without treatment; PEG and NaCL: treatments. Horizontal bars indicated comparisons between control and transformed plants due to stress. Differences between control and transformed plants under the same conditions, when significant, were displayed above the error bars. Statistical significance was assessed using a two-tailed Student’s *t*-test; * *p* < 0.5, ** *p* < 0.05, *** *p* < 0.005. ns: not significant.

Upon examining the physiological parameters of the transgenic lines *FaNAC6*-OX L23, L25 and L29, differences were found compared to the control plants. Overall, the experimental conditions of drought and salinity clearly influenced all the variables with only a few exceptions (Figure 9). Under non-stress conditions (without induced stress) transgenic plants showed higher CO_2_ assimilation rates (A) with lower intercellular CO_2_ concentration (Ci) and higher water use efficiency (WUE). When plants were subjected to drought stress, photosynthesis levels (A) show no significant differences between control and transformed plants; however, lower Ci and instantaneous quantum yield (Qy, similar in both genotypes), as well as higher WUE was observed in the *FaNAC6*-OX transgenic lines. The results obtained suggest that transgenic plants overexpressing *FaNAC6* exhibit a mechanism that allows maintaining photosynthetic activity with lower substrate concentration (Ci), resulting in an increase in WUE [124]. The lower Ci could be attributable to CO_2_ consumption, given the absence of any increase in stomatal conductance (Gs). On the other hand, the lower Qy displayed by the *FaNAC6*-OX lines is consistent with their constitutively reduced Ci: as a smaller fraction of the absorbed light energy is used in carboxylation, the operating quantum yield decreases and part of the excitation energy is re-emitted as fluorescence or channeled to alternative sinks such as photorespiration, whilst net assimilation is maintained or even enhanced [125].

Higher differences were found when plants were subjected to salinity stress. Non-transgenic plants showed a marked decrease in net assimilation in photosynthetic activity (A), almost coming to a complete halt. This stop, together with a highly significant increase in internal CO_2_ (Ci), and higher stomatal conductance (Gs) compared with transformed plants, led to a sharp decrease in water use efficiency (WUE) of control plants. On the contrary, transformed plants showed stable photosynthetic activity (A), with similar stomatal conductance (Gs) levels to the ones related to drought stress, showing a very fine water regulation mechanism that led to a very high WUE value.

Leaf vapour pressure deficit (VpdL) showed a more irregular pattern among individual plants, yet a clear decrease was observed under drought stress, whereas little to no changes were noted under salt stress; this confirms differences in regulatory mechanisms depending on the source of stress.

Therefore, physiological data indicates higher water use efficiency in *FaNAC6*-OX transgenic plants, that might be related to higher efficiency of photosynthesis under lower concentrations of intercellular CO_2_. Even considering that under water stress transgenic plants showed no differences in A and Gs compared with non-transformed ones, these control mechanisms were evidenced when intercellular CO_2_ and WUE were assessed, both with significant differences. Overall, A and Gs did not present statistical differences although it is worth mentioning that A presented marginal differences (*p* < 0.1), which together with identical Gs to control plants and lower Ci lead to significant improvement in WUE. Hence, we can conclude that *FaNAC6*-OX overexpression also helps plants facing drought stress.

### 2.10. Differential Gene Expression Analysis of N. benthamiana FaNAC6-OX Lines

**Figure A1:**
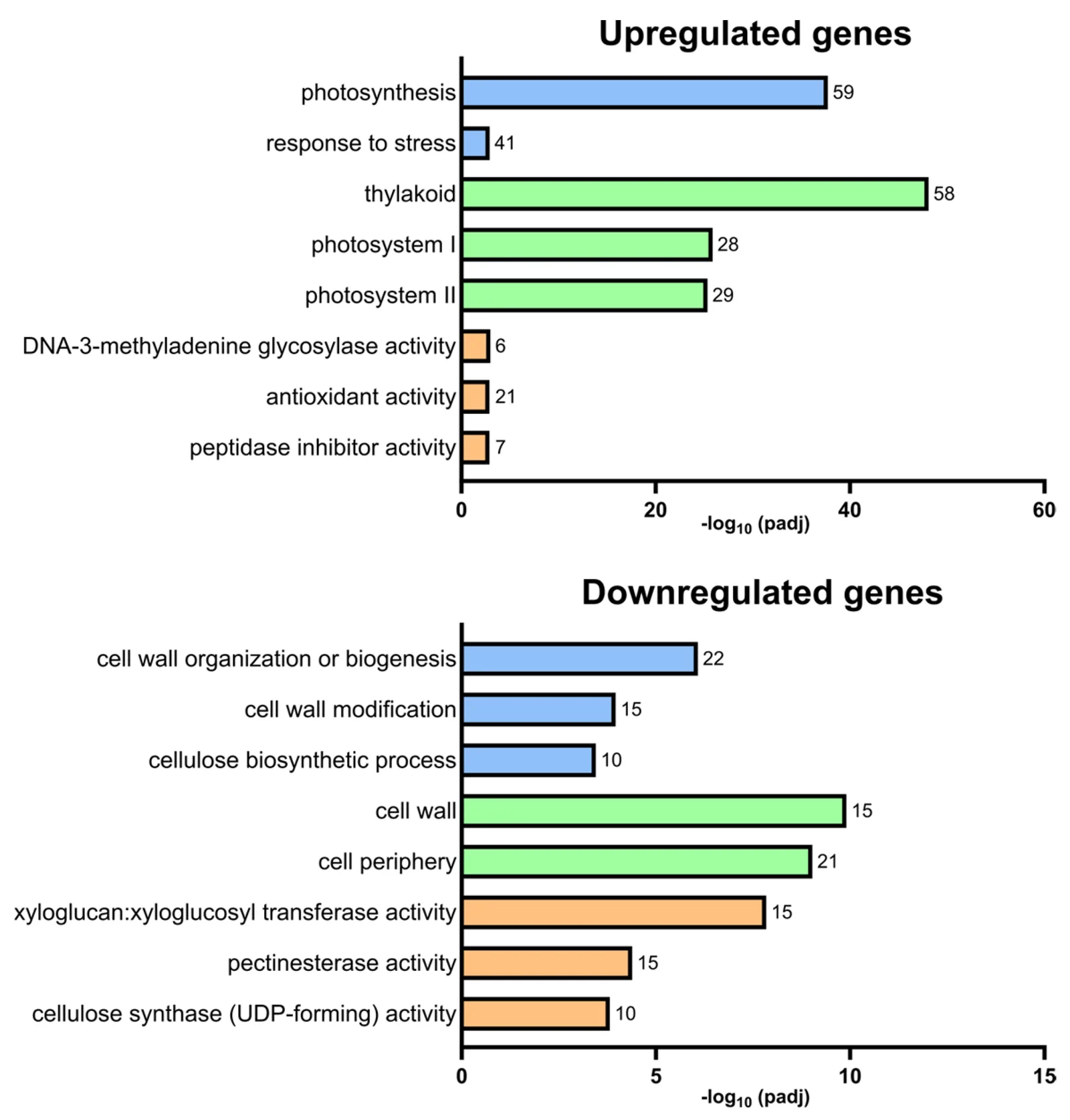
Gene Ontology (GO) enrichment analysis of upregulated and downregulated genes. Only significantly enriched (*p*adj ≤ 0.05) non-redundant GO terms were retained for visualization. Enriched terms are grouped according to the three GO categories: biological process (BP), cellular component (CC) and molecular function (MF). Bar lengths indicate enrichment significance expressed as −log10 (*p*adj), and numbers indicate the number of genes associated with each GO term. The analysis was performed using ClusterProfiler (v4.8.1).

**Figure A2:**
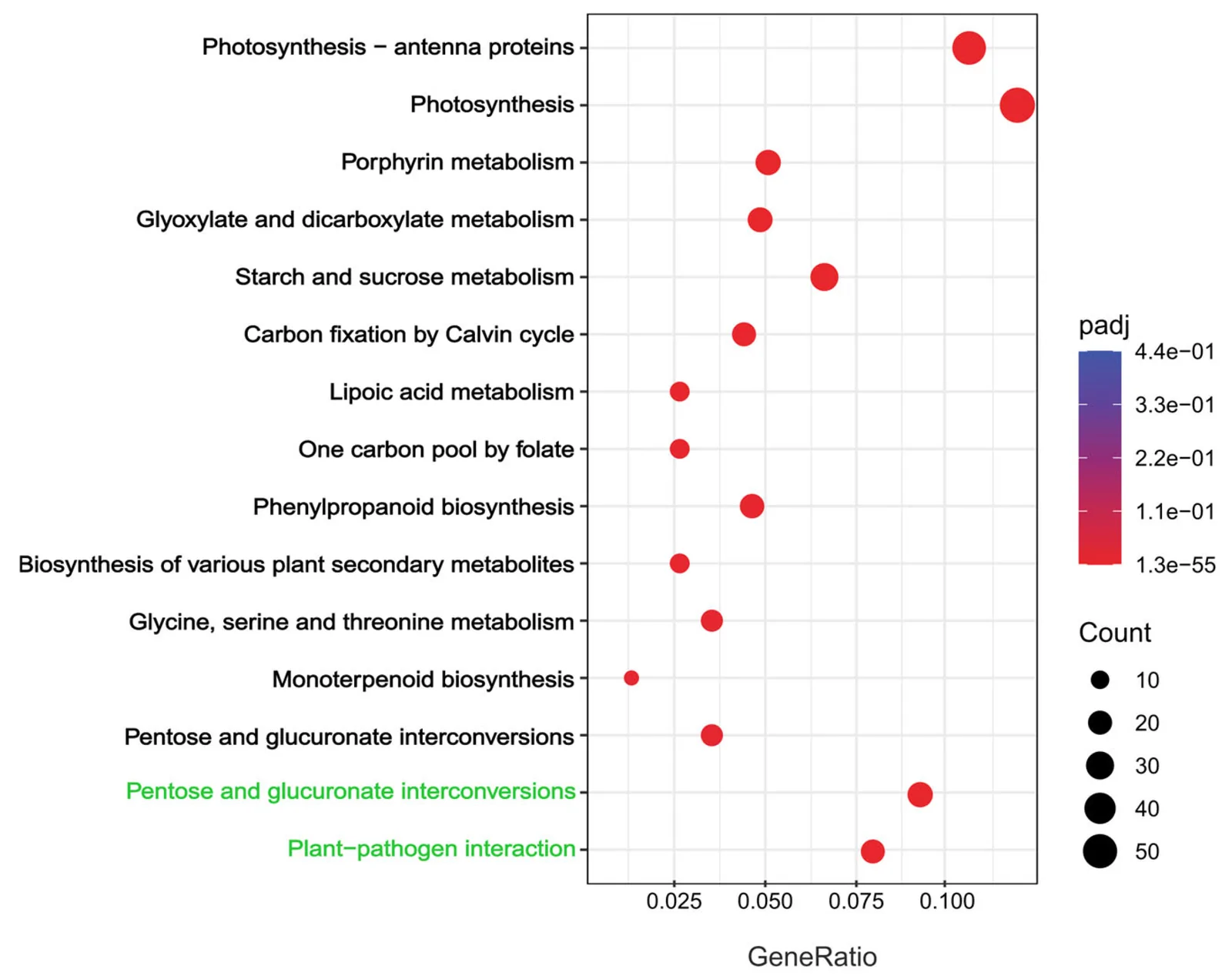
KEGG pathway enrichment analysis of differentially expressed genes (DEGs) in *N. benthamiana FaNAC6*-OX lines. Significantly enriched pathways are shown for upregulated (black) and downregulated (green) genes. Dot size represents the number of DEGs associated with each pathway, the x-axis indicates the gene ratio, and dot colour corresponds to the −log_10_ adjusted *p*-value (*p*adj). The y-axis shows the enriched KEGG pathways. The analysis was performed using ClusterProfiler (v4.8.1), with adjusted *p*-values ≤ 0.05 considered statistically significant.

To investigate the transcriptional consequences of constitutive *FaNAC6* expression under optimal growth conditions in the absence of any stress, an RNA sequencing (RNA-seq) analysis was carried out using leaves from transgenic plants (*FaNAC6*-OX) and control plants transformed with the empty vector. A preliminary analysis of the raw data, using the criteria fold change ≥ ±2 and *p*-value ≤ 0.05, identified 1.744 up-regulated genes and 1.476 down-regulated genes, whilst 36.403 genes showed no significant changes in expression compared with the controls. These results indicate a transcriptional response associated with *FaNAC6* expression in the absence of external stress. To gain a better understanding of the biological processes underlying these changes, Gene Ontology (GO) and KEGG pathway enrichment analyses were carried out using the results obtained from the previous analysis (Figure A1 and Figure A2). 

**Figure A3:**
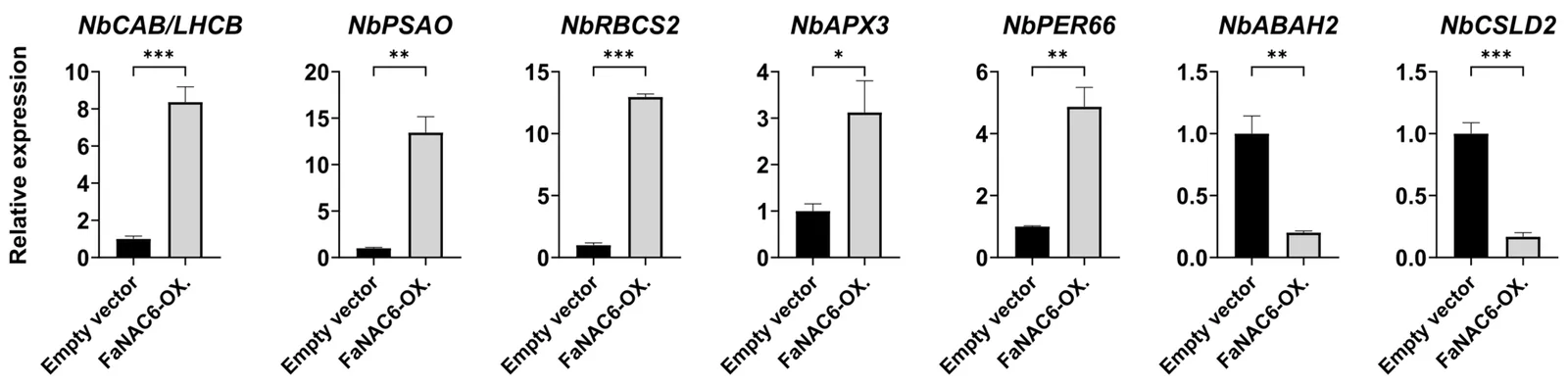
Analysis of gene expression by RT-qPCR for some of the genes showing differential expression in the transcriptomic analysis by RNA-seq of *Nicotiana FaNAC6*-OX transgenic plants. Bars show relative expression levels normalized to empty vector-transformed control plants (set to 1) and represent the mean ± SEM of three independent biological replicates. Statistical significance was determined by two-tailed Student’s *t*-test (* *p* < 0.5, ** *p* < 0.05, *** *p* < 0.005).

Subsequently, a supervised and more robust analysis of the previous results was carried out, in which only those genes with a fold change ≥ ±3, a *p*-value ≤ 0.05 and an FPKM value ≥ 5 were selected as differentially expressed genes (DEGs). A total of 748 DEGs were thus identified in *Nicotiana* plants overexpressing *FaNAC6*, of which 332 genes were up-regulated, and 416 genes were down-regulated (Table 1). These genes were grouped into different functional modules, and their identification and expression levels are specified in the Supplementary Tables S5–S8. Furthermore, the data obtained via RNA-seq were corroborated by RT-qPCR for a selected group of genes. All of these genes exhibited an expression profile similar to that previously obtained in the transcriptomic analysis (Figure A3), thereby validating the results previously obtained from the RNA-seq analysis. Overall, the results revealed transcriptional changes associated with the coordinated regulation of genes specifically related to photosynthetic performance, carbon assimilation, photorespiration, redox homeostasis, cell protection and cell wall remodelling (Figure A1 and Figure A2).

The most notable feature among the genes whose expression increased was the activation of a photosynthetic programme centred on the chloroplasts. Genes encoding components of both photosystem II and photosystem I were induced, along with several light-harvesting proteins that bind to chlorophyll a/b, including PSI-associated LHC, members of the PSII-associated LHCB/CAB family and PSAO-photosystem I subunit O gene (Supplementary Table S5). These transcripts exhibited some of the most marked changes in expression among photosynthesis-related genes, suggesting an increased light-harvesting capacity in *FaNAC6*-OX plants. Furthermore, the expression of genes encoding proteins associated with the NDH complex, such as PNSL2, was also up-regulated, indicating the activation of photosynthetic electron transport and chloroplast-associated redox processes. This coordinated induction of photosystem subunits and electron transport components is consistent with the higher net rates of CO_2_ assimilation observed in plants *FaNAC6*-OX without induced stress.

Genes involved in chlorophyll metabolism and carbon fixation were also induced (Supplementary Table S5). Among the up-regulated transcripts were genes associated with the biosynthesis or maintenance of tetrapyrrole and chlorophyll, such as HEMA1, CHLH, POR and SGRL, along with genes of the Calvin cycle, including RBCS and SBPase. The induction of these genes is consistent with the physiological phenotype observed in *FaNAC6*-OX plants, as they exhibited an increase in net CO_2_ assimilation and a reduction in intercellular CO_2_ concentration in the basal conditions. Given that stomatal conductance was not significantly altered, these results suggest that the increase in photosynthetic rate is likely associated with a greater biochemical capacity for carbon fixation rather than with changes in the regulation of stomatal control. This interpretation is supported by previous studies showing that increased activity of Calvin cycle enzymes, particularly SBPase, can enhance photosynthetic carbon assimilation and photosynthetic capacity [126,127]. Similar effects have also been reported for KfNAC83, a NAC transcription factor derived from CAM plants, whose heterologous overexpression in *Arabidopsis* increased CO_2_ assimilation, photosynthetic capacity and biomass accumulation [128].

In the context of abiotic stress, this enhanced photosynthetic and carbon-assimilation capacity would be expected to be particularly advantageous. Under drought and salinity, partial stomatal closure restricts CO_2_ entry into the leaf and lowers the intercellular CO_2_ concentration (Ci), a regime in which, according to the biochemical model of C3 photosynthesis, assimilation is limited by carboxylation capacity (Vcmax) rather than by RuBP regeneration [129]. A constitutively higher biochemical capacity for carbon fixation, resulting from the coordinated induction of Calvin cycle enzymes such as SBPase and RBCS together with the photosynthetic light reactions, would therefore enable the plant to sustain net CO_2_ assimilation despite the reduced Ci imposed by stomatal limitation, as has been reported when Calvin cycle activity is enhanced [126,127,130]. Since assimilation is maintained at a lower Ci without a concomitant increase in stomatal conductance (Gs), the CO_2_ gradient (Ca-Ci) widens and intrinsic water-use efficiency rises [124]. This framework offers a plausible explanation for the behavior of the *FaNAC6*-OX plants, which sustained net assimilation at reduced Ci and displayed higher WUE without changes in Gs under drought and, most notably, under salinity.

At the same time, the induction of genes associated with redox homeostasis and cellular protection was observed (Supplementary Table S5). Thus, the induction of several class III peroxidases, including PER12, PER42 and PER66, together with the ascorbate peroxidase APX3, has been observed, suggesting the activation of enzymatic systems involved in H_2_O_2_ detoxification and ROS homeostasis. In addition, the induction of PRO2, which encodes Δ-1-pyrroline-5-carboxylate synthetase, an enzyme involved in the proline biosynthetic pathway, was also observed, suggesting the activation of mechanisms associated with proline biosynthesis. Similarly, the induction of MLP proteins—belonging to the Bet v I protein family—has also been observed, which is consistent with a protective response to stress. The induction of all these genes to maintain redox balance is consistent with the simultaneous activation of photosynthesis and photorespiration, both of which are closely linked to this balance and to ROS production in chloroplasts. In fact, similar functions have been described for other NACs in relation to antioxidant defence, including SNAC3, TaNAC29 and ANAC013, which regulate ROS-scavenging genes and the transcriptional networks that respond to oxidative stress [71,131,132].

This expanded capacity for electron transport, photorespiration and ROS detoxification would also provide alternative sinks for the reducing power generated when carbon assimilation operates at a low Ci, as occurs under drought [133]. This is consistent with the reduced operating quantum yield (Qy) recorded in the transgenic lines under water stress, which reflects a lower photochemical demand at reduced Ci rather than a diminished photosynthetic capacity.

Furthermore, a considerable number of genes were observed to be downregulated, associated with cell wall biosynthesis and remodelling (Supplementary Table S6). Among the repressed transcripts were several xyloglucan endotransglucosylase/hydrolase genes, such as XTH2, XTH8, XTH15 and XTH16, as well as genes involved in cellulose biosynthesis, such as CESA2 and CSLD2. This pattern suggests that the overexpression of *FaNAC6* is associated with reduced expression of genes involved in primary cell wall expansion and cell wall polysaccharide remodelling. This finding is particularly interesting as it has previously been reported that several NAC transcription factors, including members of the SND, NST and VND families, act as positive regulators of secondary cell wall biosynthesis [134,135,136,137]. Therefore, *FaNAC6* appears to behave differently from other *NAC* genes, as its overexpression seems to be associated with the repression of genes related to cell wall assembly and remodelling.

**Figure 10:**
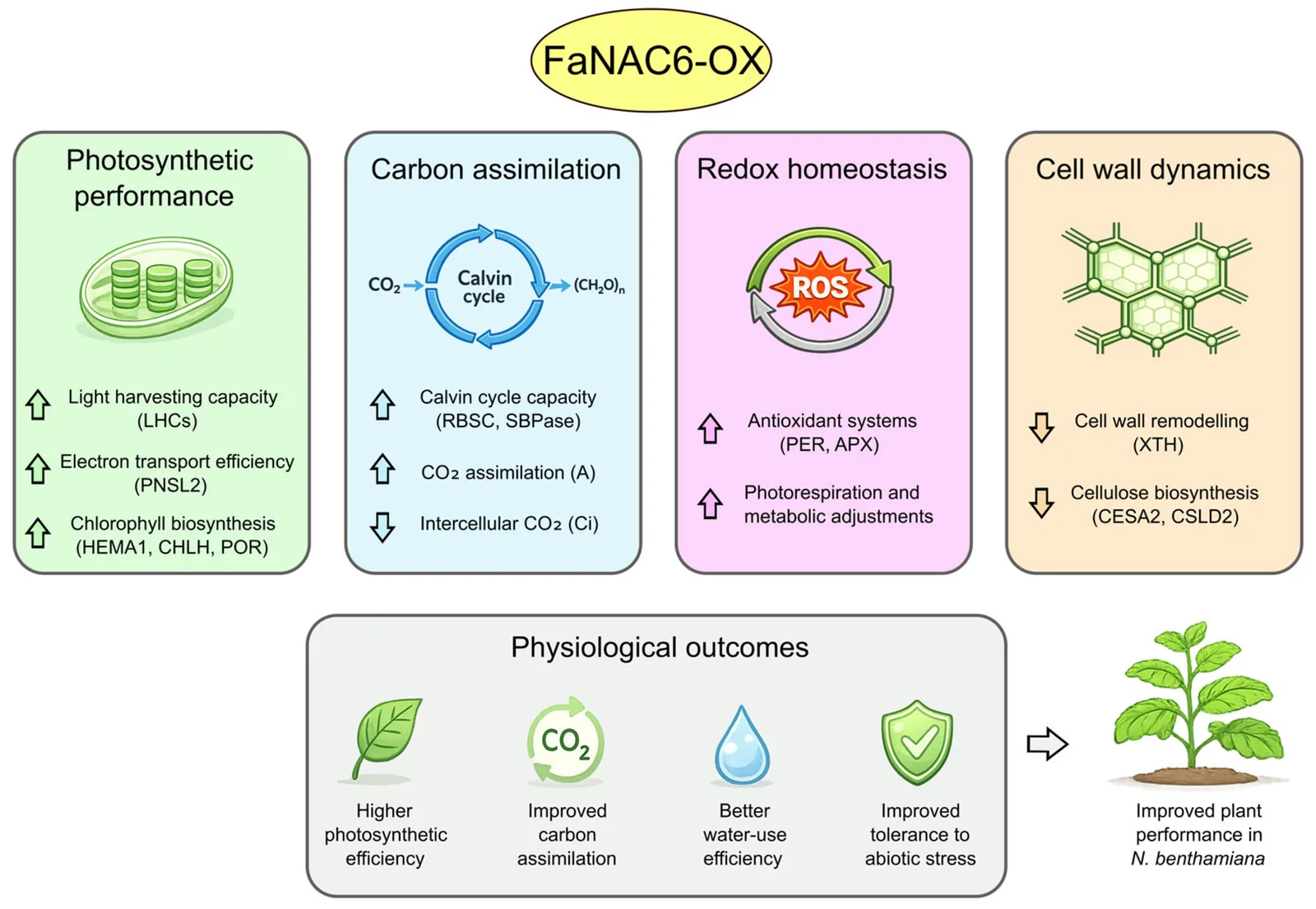
Putative model of transcriptional regulation proposed for *FaNAC6* based on transcriptomic and physiological analyses. The figure was rendered using Inkscape v1.4.1, and the illustrations were generated using artificial intelligence (OpenAI DALL·E 3).

Taken together, these results support a model whereby the overexpression of *FaNAC6* promotes a transcriptional state characterised by enhanced chloroplast function, carbon assimilation, photorespiration and redox protection, whilst repressing genes associated with cell wall expansion and structural remodelling (Figure 10). This pattern is consistent with the physiological phenotype observed in *Nicotiana FaNAC6*-OX plants, suggesting that FaNAC6 may contribute to the coordination of photosynthetic performance and cellular protection under non-stress conditions. However, these transcriptional changes should be interpreted as potential downstream responses associated with *FaNAC6* overexpression, rather than as direct transcriptional targets. Future promoter-binding or transactivation assays will be required to confirm the expression patterns of the most representative genes that appear to be directly regulated by *FaNAC6*.

## 3. Materials and Methods

### 3.1. Plant Material

The strawberry plants (*Fragaria* × *ananassa* Duch. cv. ‘Chandler’, an octoploid cultivar) utilized in this study were cultivated in a plant chamber, with a temperature maintained at 25 °C, relative humidity (RH) set at 75%, and a photoperiod of 16/8 h of light/darkness. RH was only modified during stress experiments, in which the RH value was adjusted to the design of the experiment performed. In any case, all vegetative tissues (expanded leaves and roots) utilized in subsequent analyses were collected from the plants under study, immediately frozen in liquid nitrogen, and stored at −80 °C until use.

### 3.2. Stress Treatments

The water and salt stress experiments were conducted using strawberry plants (*Fragaria* × *ananassa*, cv. ‘Chandler’) in a chamber. Prior to the initiation of the experiment, the potted plants were transferred to sterile pots containing geotextile (GEOTEXAN, S.A.U, Minas de Ríotinto, Spain) as an inert physical support, which functioned as a substrate, and 0.5× Murashige and Skoog (MS) medium (Duchefa Biochemie B.V., Haarlem, The Netherlands). Then, the plants were acclimatized for a period of 24 h at an RH of 75%.

The drought experiment was conducted with two groups of acclimatized plants subjected to different water stress conditions for a period of 5 h. In the first group of plants, root hydration was maintained but water availability in the aerial parts was restricted by reducing the environmental RH to 30%. In the second group of plants, the stress treatment was more severe, and water availability was reduced in both leaves and roots by combining 30% RH and MS medium (Duchefa Biochemie B.V., Haarlem, The Netherlands) supplemented with 20% polyethylene glycol (PEG). In both cases, the control plants were cultivated in 0.5× MS medium and 75% RH under the same conditions described above. In the salt stress experiment, acclimatized strawberry plants were also utilized, and stress was induced by adding 200 mM NaCl to the 0.5× MS medium [49]. The treatment was maintained for a period of 72 h. During the experiment, the plants were maintained at RH level of 75%. The environmental RH and temperature parameters were monitored in real time using a TRUTINA probe (ATMOS 14, METER Group, Inc., Pullman, WA, USA) during both experiments. In both situations, plant material (leaves and roots) was collected independently, frozen in liquid nitrogen, and stored at −80 °C until use.

For stress treatments in *Nicotiana benthamiana*, plants were grown individually in pots. Control plants were irrigated to runoff with 0.5× MS medium, whereas plants subjected to drought or salt stress were irrigated to runoff with 20% PEG 6000 or 200 mM NaCl, respectively, dissolved in 0.5× MS medium. Both treatments were applied for 24 h, a period selected based on the phenotypic response observed over the course of the stress treatment.

To assess transcriptional responses to signaling molecules involved in stress tolerance, plants were treated with 5-aminolevulinic acid (ALA; 10 mg L^−1^), a well-established regulator of stress resilience in strawberry [138], and sodium nitroprusside (SNP; 10 µM), used as a nitric oxide (NO) donor. Both compounds were prepared in half-strength MS medium and applied to the roots under controlled conditions. Leaf tissues were harvested for 3 days after treatment, as previously described in [95]. 

Oxidative stress was induced by foliar application of hydrogen peroxide (H_2_O_2_; 100 mM) or paraquat (PQ; 1 mM), dissolved in sterile distilled water supplemented with 0.01% (*v*/*v*) Tween-20 to ensure uniform leaf coverage. For H_2_O_2_ treatments, leaves were collected 5 h post-application, whereas PQ-treated samples were harvested at 6 h and 24 h to capture early and sustained stress responses. All experiments were conducted using three independent biological replicates.

### 3.3. Hormonal Treatments

Hormonal treatments were applied by spraying the leaves with a solution of 100 μM abscisic acid (ABA) or methyl jasmonate (MeJA) and salicylic acid (SA) at 5 mM, and ethephon at 300 mg L^−1^ in 5% (*v*/*v*) ethanol and 0.01% (*v*/*v*) Tween-20 until run-off, as previously published [139,140,141]. Control plants received the same solution without hormones. Leaf samples were collected from both treated and control plants 6 h after treatment.

### 3.4. RNA Extraction

Total RNA was isolated and purified from leaf and root samples of control strawberry plants and plants subjected to water stress and/or salt stress. Similarly, RNA was also extracted from leaves of transgenic *Nicotiana benthamiana* plants overexpressing the *FaNAC6* gene and from control plants. The Maxwell 16 Lev Plant RSC Instrument automated extraction system and the Maxwell RSC RNA FFPE kit (Promega Corporation, Madison, WI, USA) were utilized with CTAB buffer (Promega). The process was carried out in accordance with the manufacturer’s instructions. The RNA obtained was quantified with a Quantus^TM^ fluorometer using the Quantifluor^TM^ RNA kit (Promega Corporation, Madison, WI, USA). Quantification was performed following the manufacturer’s instructions. The quality and integrity of the obtained RNA was checked using an Agilent 2100 Bioanalyzer (Agilent Technologies, Santa Clara, CA, USA). 

### 3.5. Transcriptional Analysis

The selection of *FaNAC* genes showing differential expression under drought and salt stress conditions was based on previous RNA sequencing (RNA-seq) studies carried out by our research group on leaf and root tissues of strawberry plants (*Fragaria* × *ananassa*, cv ‘Chandler’) subjected to these stresses (Supplementary Table S1). The *FaNAC* genes of interest were identified using the following criteria: FPKM value > 5, fold change > ±2, and *p*-value ≤ 0.05. 

Transcriptomic analysis of transgenic *N. benthamiana* plants overexpressing *FaNAC6* (*FaNAC6*-OX) was performed using the independent transgenic lines L23, L25 and L29, which were analysed in comparison with three independent lines transformed with the empty vector (pK7WG2) (control plants). Two biological replicates for each control and transgenic line were analysed (six biological replicates for both the overexpression and control group). Library construction, sequencing and analysis were performed by Europe Novogene Technology Co., Ltd. (Cambridge, UK). Following RNA quality assessment, RNA-seq libraries were prepared from poly(A)-selected mRNA and sequenced on an Illumina platform according to the manufacturer’s standard protocols. Raw sequencing reads were aligned to the *N. benthamiana* reference genome v2.6.1 using HISAT2 (v2.2.1) software, accessed on 5 February 2026. Gene expression was quantified using FeatureCounts (v2.0.6), and expression levels were calculated as fragments per kilobase of transcript per million mapped reads (FPKM). Differential gene expression analysis was performed using DESeq2 (v1.42.0). Differential expression was calculated by expressing the log2fold values resulting of comparing normalized readings from transgenic plants against control plants. The selection of DEGs was made based on the following selection criteria: FPKM value ≥ 5, fold change ≥ ±3, and *p*-value ≤ 0.05. RNA-seq data have been deposited in the public GEO database GSE342180.

### 3.6. Bioinformatic Analysis of FaNAC Genes 

The sequences of *FaNAC* DEG were retrieved from the Phytozome BioMart module (https://phytozome-next.jgi.doe.gov/), accessed on 5 May 2025. The strawberry (*Fragaria vesca*) genome annotation v4.0 a2 from the *Rosaceae* genome database was used (GDR, https://www.rosaceae.org/), accessed on 10 May 2025. The amino acid sequences of the proteins were aligned using Clustal Omega (v 1.2.4). The Plant-mSubP software and the PseAACNCCDipep prediction module were used for subcellular location prediction [142], accessed on 24 May 2025. The identification of the conserved domain within the FaNAC proteins was conducted utilizing the online software MEME [143], accessed on 1 June 2025, with a maximum of 10 motifs and a range of motif widths from 6 to 50 as parameters.

### 3.7. Phylogenetic Analysis and Motifs of FaNAC Genes

Phylogenetic tree, including differentially expressed FaNAC proteins over published NACs and *Arabidopsis thaliana* NAC (AtNAC) family members, were both conducted using MEGA 12 software [144], accessed on 10 June 2025. Neighbor-joining statistical method and a bootstrap of 1000 replicates were applied. The sequences of AtNAC proteins were obtained from the Phytozome14 database (https://phytozome-next.jgi.doe.gov/, accessed on 5 June 2025).

### 3.8. Synteny Analysis 

The analysis was conducted utilizing the genomes of *F. vesca* v4.0 a2 and *Fragaria* × *ananassa* Royal Royce v1.0, which were obtained from the *Rosaceae* genome database (GDR, https://www.rosaceae.org/, accessed on 6 June 2025). A comparison and processing of both genomes was conducted using the Multiple Covariance Scanning Toolkit (MCScanX), with an E-value threshold of 1 × 10^−10^ [145], accessed on 8 June 2025. The collinearity analysis was visualized using the TBTools-II v2.154 program [146], accessed on 12 June 2025.

### 3.9. Promoter Analysis 

The 2000 bp upstream sequences of the start codon (ATG) of the *FaNAC* target genes were obtained from the *F. vesca* v4.0 a2 genome extracted from the Phytozome database and analyzed with PlantCARE (http://bioinformatics.psb.ugent.be/webtools/plantcare/html/, accessed on 19 July 2025) to identify potential cis-regulatory elements. Raw data management and rendering were done using Python 3.11.0 with Pandas and Matplotlib libraries (v 3.7.1), accessed on 22 July 2025. The final figures were prepared with Inkscape v1.4 software, accessed on 30 July 2025.

### 3.10. Gene Expression Analysis by RT-qPCR of FaNAC Genes

The validation of the expression data obtained in the transcriptomic analysis by RNA-seq was carried out by RT-qPCR using a CFX real-time PCR system (Bio-Rad, Hercules, CA, USA) and specific primers for the selected *FaNAC* genes (Supplementary Table S9). To ensure the reliability of the results, at least three biological and technical replicates of each tissue type and experimental condition were utilized for the isolation and purification of total RNA. The Bio-Rad iScript Reliance Select kit (Bio-Rad, Hercules, CA, USA) was employed for the synthesis of cDNA, with 2 µg of total RNA being used in each reaction. For quantitative polymerase chain reaction (qPCR), the BioRAD SsoAdvanced Universal SYBRGreen Supermix kit was utilized. Amplification was performed on 25 ng of cDNA obtained for each of the samples under study. Expression analysis of genes of interest was performed in triplicate for each sample analyzed and the specificity of the amplification was verified by analyzing the melting curves. Relative expression values were calculated according to the ΔΔCt method [147] using the constitutive genes *FaGADPH2* and *FaEF1α* for data normalization [148].

The validation of the results obtained from RNA-seq of the *N. benthamiana FaNAC6*-OX transgenic plants, and their analysis were carried out using RT-qPCR in the same manner as described above. For this purpose, specific primers designed from the sequences of selected *Nicotiana* DEGs were used (Supplementary Table S9). In this case, the housekeeping gene used for data normalisation was the ubiquitin gene (*NbUBQ3*). 

### 3.11. Generation of Nicotiana benthamiana Plants Overexpressing FaNAC6

The full-length CDS of the *FaNAC6* gene (*FvH4_3g20690*) was obtained from the Phytozome14 database, accessed on 30 July 2025. This sequence was synthesized using synthetic biology (GenScript, Piscataway, NJ, USA) and inserted into the pK7WG2 overexpression vector (https://vectorvault.vib.be/, accessed on 30 July 2025) containing the CaMV35S promoter to generate the pK7WG2::*FaNAC6*-OX construct (*FaNAC6*-OX). This construct and the empty vector pK7WG2 were used to generate *FaNAC6*-OX transgenic plants and control plants via *Agrobacterium*-mediated stable transformation of tobacco (*Nicotiana benthamiana*) leaves using *Agrobacterium tumefaciens* strain GV310, following the protocol described by [149]. The resulting plants were acclimatized and transferred to pots. We used RT-qPCR to identify the *FaNAC6* overexpression lines.

### 3.12. Determination of Physiological Parameters of Nicotiana FaNAC6-OX Transgenic Plants 

To assess the physiological response of *Nicotiana FaNAC6*-OX plants, three independent transgenic lines (L23, L24 and L29), representing distinct transformation events, were evaluated alongside control plants carrying the empty vector (two independent lines). Six biological replicates were measured per line. Gas exchange measurements were performed on the youngest fully expanded leaves using a LI-6400/XT Portable Photosynthesis System (LI-COR Biosciences, Lincoln, NE, USA) equipped with the 6400-01 CO_2_ injector and the leaf chamber fluorometer 6400-40 LCF. Parameters were set at 400 µmol mol^−1^ CO_2_, flow of 350 µmol s^−1^ and PAR in 1000 µmol m^−2^ s^−1^.

Three technical repetitions were measured from each biological replicate, allowing a stabilization time of 75 seconds for each measurement. Following, net assimilation (A, μmol CO_2_ m^−2^ s^−1^), stomatal conductance (Gs, molH_2_O m^−2^ s^−1^), intercellular CO_2_ concentration (Ci, µmol CO_2_ mol CO_2_^−1^), instantaneous quantum yield (Qy, dimensionless), vapour pressure deficit in leaves (VpdL, kPa) and water use efficiency (WUE, μmol CO_2_ mol^−1^ H_2_O) were calculated and analyzed. For the statistical analysis, normal distribution of the data was checked using the Anderson-Darling test. Then, variables which did not fit normal distribution were log- transformed for statistical analysis and variance was assessed using ANOVA test. Differences between means were checked by means of the Tukey post hoc test for the least significance difference (LSD) threshold. Significance level was established in *p* = 0.05 for all the statistical procedures. The analysis of all the physiological variables was carried out in the R environment [150] using the RStudio user interface (v 2025.05.1) [151].

## 4. Conclusions

This study presents a comprehensive genomic and transcriptomic analysis of the *FaNAC* genes that respond differentially to drought and salinity in the strawberry (*Fragaria* × *ananassa*) and identifies *FaNAC6* as a potential regulatory gene governing the strawberry plant’s response to these abiotic stresses. Transcriptomic analysis using RNA-seq, followed by validation of these results via RT-qPCR, has enabled us to identify thirty-five *FaNAC* genes with differential expression under drought and salinity conditions in strawberry, some of which have shown tissue-specific and/or abiotic stress-specific expression. Indeed, whilst some *FaNAC* genes were significantly up-regulated in leaves and roots subjected to drought and salinity, others were specifically up-regulated in a particular tissue or in response to a specific type of stress, suggesting that these *FaNAC* genes may be involved in a highly specialized regulatory network, rather than in a homogeneous and generalized response to abiotic stress. From a phylogenetic perspective, these *FaNAC* genes showed high homology with orthologous genes from *Arabidopsis*, whilst their collinearity analysis revealed a high degree of conservation in the distribution of these genes across the subgenomes of *F.* × *ananassa*. Analysis of its promoters predicted the presence of cis-regulatory elements associated with hormonal and stress responses, particularly linked to ABA and MeJA, indicating their potential involvement in complex networks of transcriptional regulation mediated by abiotic signals. *FaNAC6* was selected for further molecular characterization based on significant induction of expression in both the leaves and roots of strawberry plants subjected to both drought and salinity. Furthermore, *FaNAC6* showed induced expression under conditions of oxidative stress and in the presence of ABA. Its overexpression in *Nicotiana benthamiana* enhanced the stress tolerance of the resulting transgenic plants by improving their photosynthetic rate (A) and water use efficiency (WUE). Simultaneously, transcriptomic analysis of the aforementioned plants revealed the induction of genes associated with photosynthesis (e.g., LHCS, PNSL2, POR, etc.), carbon assimilation (e.g., RBSC, SBPase, etc.), and redox homeostasis (e.g., PER, APX, etc.), while genes associated with cell wall expansion and structural remodeling (e.g., XTH, CSLD2, etc.) were repressed. These results provide a comprehensive explanation of the physiological outcomes and postulate *FaNAC6* as a putative gene regulating photosynthetic performance and cell protection under non-stress conditions in strawberry leaves. 

## Figures and Tables

**Table 1:** Putative functional modules and the up- and down-regulated number of genes included in each of them.

Putative Functional Module	UP Genes	DOWN Genes	Total	Functional Interpretation
Carbon assimilation, photorespiration and energy metabolism	17	11	28	Mostly UP/mixed: Calvin cycle, photorespiration, sugar and energy metabolism; compatible with enhanced biochemical capacity and C/N integration.
Cell wall, cuticle and cell-surface remodeling	29	50	79	Strong DOWN component plus selected UP genes; suggests repression of wall/cuticle expansion with selective remodeling.
Dehydration/stress-protective proteins	1	4	5	Mostly DOWN: LEA/dehydrin/ERD-like genes; suggests no broad activation of classical dehydration-protection genes.
Hormone, growth and developmental regulation	13	34	47	More DOWN than UP: hormone, growth, senescence/developmental regulators; suggests modulation of growth programmes.
Membrane, Ca^2+^/osmotic signaling and transport	49	51	100	Prominent in both UP and DOWN: ion/nutrient transporters, Ca^2+^/membrane-associated signaling and trafficking.
Other metabolic or cellular processes	31	82	113	Heterogeneous enzymes/cellular functions.
Photosynthesis/chloroplast function	87	3	90	Predominantly UP: light-harvesting, photosystem and chlorophyll-related genes; supports activation of photosynthetic machinery.
Redox homeostasis and oxidative-stress responses	20	13	33	Mostly UP/mixed: peroxidases, APX/redox-related enzymes; suggest ROS/redox adjustment.
Regulatory proteins, RNA/protein homeostasis and stress regulators	17	57	74	Mostly DOWN: TFs, RNA/protein turnover and stress regulatory nodes, including canonical stress regulators.
Specialized metabolism and stress-associated secondary metabolism	8	14	22	Mixed: phenylpropanoid/terpenoid/flavonoid/lipid-derived metabolism.
Stress/defense-associated proteins and protease/protease-inhibitor responses	14	7	21	Mostly UP: protease inhibitors, PR/MLP/wound-associated and protease-related proteins.
Unknown or poorly annotated genes	46	90	136	No-description/DUF/poorly annotated genes.
	332	416	748	

## Data Availability

The original contributions presented in this study are included in the article/Supplementary Materials. Further inquiries can be directed at the corresponding author. RNA-seq data have been deposited in the public GEO database GSE342180.

## Supplementary Materials

The following supporting information can be downloaded at https://www.mdpi.com/article/10.3390/ijms27167352/s1.

## Abbreviations

The following abbreviations are used in this manuscript:

TF	Transcription factor
ROS	Reactive oxygen species
RH	Relative humidity
PEG	Polyethylene glycol
PQ	Paraquat
SNP	Sodium nitropruside
ABA	Abscisic acid
ALA	5-Aminolvulinic acid
MeJA	Methyl jasmonate
SA	Salicylic acid
NO	Nitric oxide
