# Supplementary material for: Integrated Genomic and Functional Analyses Identify FaNAC6 as a Candidate Regulator of Drought and Salinity Responses in Strawberry (Fragaria × ananassa)
Source: Int J Mol Sci. 2026 Aug 17;27(16):7352. doi: 10.3390/ijms27167352 (PMC13513608; doi:10.3390/ijms27167352)

Integrated genomic and functional analyses identify *FaNAC6* as a candidate regulator of drought and salinity responses in strawberry (*Fragaria x ananassa*)

Supplementary Tables S1-S4 and Figures S1 and S2

Supplementary Table S1. *FaNAC* genes with differential expression in leaves and/or roots of strawberry plants in response to drought and salinity determined by RNAseq and RT-qPCR. 'No var' indicates that there is no variation in expression. All these genes showed fold change (FC) values ≥ ± 2, p-value ≤ 0.001, and FPKM ≥ 5. DL: drought in leaves; DR: drought in roots; SL: salinity in leaves; SR: salinity in roots.

			Fold Change RNAseq				Fold Change qRT-PCR				
			Drought		Salinity		Drought		Salinity		
Expression Pattern	Gene name	NAC Name [44]	Leaf	Root	Leaf	Root	Leaf	Root	Leaf	Root	Description
**GROUP 1: UP** (DL, DR, SL, SR)	*FaNAC1*	*FvNAC079*	22,63	No var.	33,82	30,91	44,72	5,70	23,61	36,33	Q9FMR3∣NAC90_ARATH NAC domain-containing protein 90
	FaNAC3	*FvNAC004*	4,08	2,04	40,79	12,73	7,63	3,20	35,59	12,48	Q8H115∣NA102_ARATH NAC domain-containing protein 102
	FaNAC5	*FvNAC015*	9,58	2,38	64,89	12,91	17,26	2,69	21,73	14,50	Q39013∣NAC2_ARATH NAC domain-containing protein 2
	FaNAC6	*FvNAC034*	59,30	3,34	56,89	35,75	46,30	4,42	30,65	39,30	A0A3Q7HH64∣JA2L_SOLLC NAC domain-containing protein JA2L
	FaNAC8	*FvNAC045*	63,12	3,41	30,91	27,67	16,67	4,15	80,08	30,78	K4BWV2∣NAP1_SOLLC NAC domain-containing protein 1
	FaNAC10	*FvNAC085*	15,14	No var.	23,10	4,72	10,74	4,15	31,87	5,00	Q9SK55∣NAC42_ARATH Transcription factor JUNGBRUNNEN 1
	FaNAC12	*FvNAC008*	2,81	No var.	5,46	4,76	5,33	2,98	7,79	7,25	Q9XIC5∣NAC17_ARATH NAC domain-containing protein 17
	FaNAC16	*FvNAC035*	6,11	No var.	12,30	14,22	10,20	5,24	5,81	22,49	Q9LD44∣NAC56_ARATH NAC transcription factor 56
	FaNAC20	*FvNAC055*	15,24	No var.	10,20	2,01	5,78	2,77	11,33	3,64	Q9FLJ2∣NC100_ARATH NAC domain-containing protein 100
	FaNAC29	…	No var.	No var.	No var.	3,23	6,39	2,26	2,31	4,11	Q94F58∣NAC89_ARATH NAC domain-containing protein 89
	FaNAC92	*FvNAC092*	15,67	3,03	3,03	13,83	27,05	3,80	42,46	15,02	Q9FLJ2∣NC100_ARATH NAC domain-containing protein 100
**GROUP 2:** UP (DL, SL, SR)	*FaNAC2*	…	3,39	No var.	5,78	11,39	9,52	No var.	4,97	13,66	F4JN35∣NTL9_ARATH Protein NTM1-like 9
	FaNAC14	*FvNAC016*	2,83	No var.	5,70	5,94	4,28	No var.	5,45	6,96	Q93VY3∣NAC72_ARATH NAC domain-containing protein 72
	FaNAC17	…	4,76	No var.	8,75	16,45	5,81	No var.	3,02	12,55	Q84K00∣NAC78_ARATH NAC domain-containing protein 78
**GROUP 3: UP** (DR, SL, SR)	*FaNAC22*	*FvNAC023*	No var.	No var.	147,03	No var.	No var.	4,44	723,41	3,15	Q9SK55∣NAC42_ARATH Transcription factor JUNGBRUNNEN 1
	FaNAC26	*FvNAC006*	No var.	No var.	9,85	183,55	No var.	2,01	26,57	193,18	Q9FK44∣NAC87_ARATH NAC domain-containing protein 87
**GROUP 4: UP** (DL, DR, SR)	*FaNAC21*	*FvNAC050*	2,53	No var.	No var.	No var.	4,65	2,22	No var.	2,32	PTHR31744:SF229 - NAC TRANSCRIPTION FACTOR
**GROUP 5: UP** (DL, DR, SL)	*FaNAC9*	*FvNAC042*	5,39	No var.	5,74	No var.	13,08	2,15	4,98	No var.	Q9FY93∣NAC83_ARATH NAC domain-containing protein 83
**GROUP 6: UP** (DL, SR)	*FaNAC4*	*FvNAC011*	3,78	No var.	No var.	4,00	11,05	No var.	No var.	4,15	PTHR31744:SF230 - NAC DOMAIN-CONTAINING PROTEIN
	FaNAC13	*FvNAC018*	5,28	No var.	No var.	4,53	5,56	No var.	No var.	3,72	PTHR31719:SF236 - NAC DOMAIN-CONTAINING PROTEIN
**GROUP 7: UP** (SL, SR)	*FaNAC24*	*FvNAC096*	No var.	No var.	2,93	6,63	No var.	No var.	3,00	7,06	Q9SQX9∣NAC50_ARATH NAC domain containing protein 50
**GROUP 8: UP** (DL)	*FaNAC11*	…	4,38	No var.	6,15	No var.	7,10	No var.	No var.	No var.	Q9LD44∣NAC56_ARATH NAC transcription factor 56
	FaNAC15	*FvNAC027*	12,21	No var.	No var.	No var.	8,44	No var.	No var.	No var.	PTHR31744:SF96 - NAC DOMAIN SUPERFAMILY
**GROUP 9: UP** (SR)	*FaNAC23*	*FvNAC010*	No var.	No var.	2,33	No var.	No var.	No var.	No var.	3,57	Q9FY82∣NAC82_ARATH NAC domain-containing protein 82
	FaNAC28	*FvNAC100*	No var.	No var.	No var.	5,94	No var.	No var.	No var.	8,82	Q84WP6∣NAC43_ARATH NAC domain-containing protein 43
**GROUP 10: UP** (SL, SR) / **DOWN** (DL)	*FaNAC25*	*FvNAC022*	-5,34	No var.	4,44	11,00	-3,44	No var.	9,12	21,84	O49459∣NAC73_ARATH NAC domain-containing protein 73
**GROUP 11: UP** (DL, SR) / **DOWN** (DR, SL)	*FaNAC18*	*FvNAC003*	2,64	-3,61	No var.	No var.	8,49	-2,04	-65,78	12,55	PTHR31744:SF214 - NAC DOMAIN-CONTAINING PROTEIN
**GROUP 12: UP** (DL) / **DOWN** (SL, SR)	*FaNAC38*	*FvNAC057*	No var.	-3,31	No var.	-2,30	2,26	No var.	-4,16	-7,69	O49697∣NAC71_ARATH NAC domain-containing protein 71
	FaNAC40	*FvNAC014*	No var.	No var.	-14,85	No var.	3,08	No var.	-10,00	-2,12	PF02365 - No apical meristem (NAM) protein (NAM)
**GROUP 13: UP** (DR) / **DOWN** (SL)	*FaNAC31*	*FvNAC021*	-2,7	No var.	-8,4	No var.	No var.	2,69	-3,22	No var.	Q8GY42∣NAC25_ARATH NAC transcription factor 25
**GROUP 14: UP** (SR) / **DOWN** (SL)	*FaNAC27*	*FvNAC105*	No var.	No var.	No var.	17,4	No var.	No var.	-2,43	12,39	Q9LVA1∣NC101_ARATH NAC domain-containing protein 101
**GROUP 15: DOWN** (DL, DR, SL, SR)	*FaNAC35*	*FvNAC049*	No var.	-15,9	No var.	-12,6	-5,55	-2,17	-11,10	-5,00	Q9FY93∣NAC83_ARATH NAC domain-containing protein 83
**GROUP 16: DOWN** (DL, SL)	*FaNAC33*	*FvNAC061*	-3,78	No var.	-2,91	No var.	-5,00	No var.	-2,12	No var.	Q9ZVP8∣NAC35_ARATH NAC domain-containing protein 35
**No var.**	*FaNAC37*	*FvNAC083*	No var.	-3,57	No var.	No var.	No var.	No var.	No var.	No var.	O49459∣NAC73_ARATH NAC domain-containing protein 73
	FaNAC39	…	No var.	-2,31	No var.	No var.	No var.	No var.	No var.	No var.	Q9FWX2∣NAC7_ARATH NAC domain-containing protein 7

Supplementary Table S2. Results of domains prediction obtained with InterProScan online software

Protein	Structural domains	Entry	Name	Start	End	e-value
**FaNAC1**	Pfam	PF02365	No apical meristem (NAM) protein	5	143	1.8E-33
**FaNAC2**	Pfam	PF02365	No apical meristem (NAM) protein	14	139	7.4E-43
**FaNAC3**	Pfam	PF02365	No apical meristem (NAM) protein	8	131	7.9E-45
**FaNAC4**	Pfam	PF02365	No apical meristem (NAM) protein	12	138	3.8E-41
**FaNAC5**	Pfam	PF02365	No apical meristem (NAM) protein	8	131	3.3E-46
**FaNAC6**	Pfam	PF02365	No apical meristem (NAM) protein	15	139	6.7E-44
**FaNAC8**	Pfam	PF02365	No apical meristem (NAM) protein	10	134	1.5E-48
**FaNAC9**	Pfam	PF02365	No apical meristem (NAM) protein	15	138	6.8E-44
**FaNAC10**	Pfam	PF02365	No apical meristem (NAM) protein	22	154	2.1E-35
**FaNAC11**	Pfam	PF02365	No apical meristem (NAM) protein	5	140	5.5E-23
	Pfam	PF02365	No apical meristem (NAM) protein	319	432	1.5E-9
**FaNAC12**	Pfam	PF02365	No apical meristem (NAM) protein	19	145	1.4E-42
**FaNAC13**	Pfam	PF02365	No apical meristem (NAM) protein	11	117	1.4E-12
**FaNAC14**	Pfam	PF02365	No apical meristem (NAM) protein	12	147	1.0E-18
**FaNAC15**	Pfam	PF02365	No apical meristem (NAM) protein	12	135	2.1E-40
**FaNAC16**	Pfam	PF02365	No apical meristem (NAM) protein	19	150	6.1E-48
**FaNAC17**	Pfam	PF02365	No apical meristem (NAM) protein	11	136	3.5E-44
**FaNAC18**	Pfam	PF02365	No apical meristem (NAM) protein	9	136	1.9E-45
**FaNAC20**	Pfam	PF02365	No apical meristem (NAM) protein	24	148	9.8E-47
**FaNAC21**	Pfam	PF02365	No apical meristem (NAM) protein	10	143	5.7E-44
**FaNAC22**	Pfam	PF02365	No apical meristem (NAM) protein	25	153	6.2E-42
**FaNAC23**	Pfam	PF02365	No apical meristem (NAM) protein	7	133	8.4E-41
**FaNAC24**	Pfam	PF02365	No apical meristem (NAM) protein	26	151	8.3E-43
**FaNAC25**	Pfam	PF02365	No apical meristem (NAM) protein	63	202	2.4E-23
**FaNAC26**	Pfam	PF02365	No apical meristem (NAM) protein	20	144	6.9E-46
**FaNAC27**	Pfam	PF02365	No apical meristem (NAM) protein	9	163	3.2E-15
**FaNAC28**	Pfam	PF02365	No apical meristem (NAM) protein	16	143	4.6E-43
**FaNAC29**	Pfam	PF02365	No apical meristem (NAM) protein	131	254	6.9E-43
**FaNAC31**	Pfam	PF02365	No apical meristem (NAM) protein	16	142	1.6E-47
**FaNAC33**	Pfam	PF02365	No apical meristem (NAM) protein	36	160	1.7E-41
**FaNAC35**	Pfam	PF02365	No apical meristem (NAM) protein	15	138	1.6E-41
**FaNAC38**	Pfam	PF02365	No apical meristem (NAM) protein	7	133	1.8E-44
**FaNAC40**	Pfam	PF02365	No apical meristem (NAM) protein	11	137	3.0E-40
**FaNAC92**	Pfam	PF02365	No apical meristem (NAM) protein	17	141	8.2E-47

Supplementary Table S3. Prediction of the subcellular localisation of FaNAC proteins with differential expression under drought and/or salinity conditions. Nuclear localization signal (NLS) prediction results obtained with the Localizer online software (https://localizer.csiro.au/). Pred: prediction.

	Pred	Memb	Celwall	Cyto.Golgi	Cyto.Nucl	Cyto	Endopl	Ext.cel	Golgi	Mito.Plast	Mito	Nucl	Per	Plast	Vac
FaNAC1	**Nucl**	0.5%	0.063%	0.55%	1.3%	7.5%	0.2%	0.49%	0.65%	0.21%	0.4%	**85%**	0.31%	1.2%	1.3%
FaNAC2	**Nucl**	20%	0.54%	0.43%	1.6%	10%	8.4%	1.2%	9.5%	0.9%	1.4%	**35%**	1.5%	7.1%	2.5%
FaNAC3	**Nucl**	6.3%	0.89%	0.95%	4.6%	4.6%	2.8%	5.9%	0.68%	0.36%	3.5%	**50%**	0.72%	7.5%	11%
FaNAC4	**Nucl**	6.4%	0.32%	0.21%	3.8%	13%	2%	0.18%	3.6%	0.8%	1.3%	**62%**	0.48%	5.1%	0.69%
FaNAC5	**Nucl**	1.9%	0.35%	0.74%	2.3%	8.7%	1.9%	2.8%	1.3%	0.52%	4.9%	**54%**	9.5%	7.7%	3.9%
FaNAC6	**Nucl**	2.5%	0.13%	0.18%	1.1%	2%	0.78%	0.46%	0.34%	0.16%	0.28%	**87%**	0.87%	3.3%	0.53%
FaNAC8	**Nucl**	1.8%	6%	0.45%	6.4%	3.6%	0.93%	1.3%	2%	0.21%	0.66%	**68%**	1.1%	5.2%	2.4%
FaNAC9	**Nucl**	3%	0.18%	0.15%	3.6%	1.9%	1%	0.069%	0.98%	0.21%	0.61%	**76%**	0.4%	11%	0.96%
FaNAC10	**Nucl**	3.4%	0.084%	0.46%	6%	7.5%	1.5%	0.76%	0.3%	0.064%	0.51%	**74%**	0.13%	4.8%	0.5%
FaNAC11	**Plast**	2.8%	0.15%	0.32%	0.85%	11%	1.3%	0.65%	1%	0.99%	1.2%	27%	3.2%	**48%**	0.78%
FaNAC12	**Nucl**	36%	0.23%	0.96%	2.8%	5.6%	1.5%	0.55%	6.7%	0.54%	0.6%	**38%**	1.9%	3.9%	0.51%
FaNAC13	**Nucl**	0.62%	1.1%	0.18%	4.7%	4.5%	0.88%	2.2%	0.54%	0.14%	0.66%	**82%**	0.38%	0.73%	1%
FaNAC14	**Nucl**	2.1%	0.22%	0.26%	5.8%	6.2%	1.9%	0.5%	2.3%	0.93%	2.5%	**61%**	0.79%	9.2%	6.5%
FaNAC15	**Nucl**	2.5%	0.46%	0.73%	7.4%	20%	3.3%	2%	3.5%	0.32%	1.5%	**49%**	1.2%	6.4%	2.1%
FaNAC16	**Nucl**	19%	0.43%	0.31%	4%	5.5%	0.52%	0.78%	5.1%	0.15%	0.31%	**54%**	4.8%	3.9%	0.82%
FaNAC17	**Cyto**	4.6%	0.48%	0.14%	2.1%	**37%**	4.5%	0.79%	16%	0.86%	0.94%	6.3%	4.3%	21%	0.98%
FaNAC18	**Nucl**	2.5%	0.1%	0.31%	29%	5.8%	0.43%	0.31%	0.76%	0.15%	0.45%	**56%**	0.57%	2.1%	1.1%
FaNAC20	**Nucl**	1%	0.076%	0.45%	1.5%	7.5%	0.18%	0.19%	0.1%	0.026%	0.24%	**87%**	0.32%	1%	0.36%
FaNAC21	**Nucl**	5.6%	0.49%	0.38%	0.62%	17%	5.2%	1.4%	15%	1.6%	0.8%	**28%**	2.2%	20%	2.8%
FaNAC22	**Nucl**	0.93%	0.38%	0.32%	1.6%	5.2%	0.26%	0.28%	2.4%	0.053%	0.12%	**87%**	0.21%	1.2%	0.15%
FaNAC23	**Cyto**	0.46%	0.13%	1.5%	5.3%	**44%**	0.51%	0.36%	1.2%	0.22%	0.41%	40%	0.44%	4.7%	0.28%
FaNAC24	**Nucl**	2%	0.32%	0.34%	20%	8.3%	0.99%	0.66%	0.76%	1.9%	3.3%	**31%**	0.42%	29%	1.2%
FaNAC25	**Nucl**	1.6%	0.1%	0.069%	25%	24%	0.53%	0.14%	0.42%	0.11%	0.51%	**43%**	3%	0.92%	0.55%
FaNAC26	**Nucl**	0.56%	0.054%	0.11%	1.7%	15%	0.13%	0.16%	0.22%	0.03%	0.21%	**81%**	0.085%	0.66%	0.26%
FaNAC27	**Nucl**	0.95%	0.14%	0.8%	18%	9%	1.1%	0.19%	1.8%	0.063%	0.12%	**65%**	0.7%	0.63%	1.8%
FaNAC28	**Nucl**	1.5%	0.18%	0.21%	25%	10%	0.31%	0.25%	0.66%	0.22%	0.5%	**58%**	0.8%	2%	0.46%
FaNAC29	**Nucl**	12%	1.4%	0.24%	9.8%	4.3%	1.9%	0.26%	12%	0.26%	2%	**44%**	1.7%	9.1%	0.59%
FaNAC31	**Nucl**	6.2%	0.15%	0.084%	6.4%	1.8%	0.11%	0.16%	2.3%	0.065%	0.061%	**80%**	0.9%	1.3%	0.22%
FaNAC33	**Nucl**	0.88%	0.059%	0.31%	21%	3.5%	0.39%	0.16%	1.3%	0.068%	0.22%	**70%**	0.32%	0.69%	0.85%

Supplementary Table S4. *NAC* genes orthologous to *FaNAC6* described in different higher plants and related to the response to abiotic stresses

GENE	ID	SPECIES	FUNCTION	REFERENCE
RcNAC72	XP_024156501.1	*Rosa chinensis*	Positive regulator of drought tolerance, ABA sensitivity in overexpression	[123]
VvNAC17	GSVIVT01014403001	*Vitis vinifera*	Positive regulator of drought, cold and salt tolerance	[122]
SlNAC10	KJ933532.1	*Suaeda liaotungensis*	Involved in proline synthesis, positive regulator of drought and salt tolerance	[121]
GmSIN1	KY661969.1	*Glycine max*	Positive regulator of salt tolerance	[119]
AhNAC2	EU755023	*Arachis hypogaea*	Positive regulator of drought and salt tolerance	[118]
TsNAC1	AK352535.1	*Thellungiella halophile*	Positive regulator of thermotolerance, salt and drought tolerance	[117]
RD26 (ANAC072)	AT4G27410	*Arabidopsis thaliana*	Positive regulator of drought response	[116]
ANAC055	AT3G15500	*Arabidopsis thaliana*	Positive regulator of drought tolerance	[21]
ANAC019	AT1G52890	*Arabidopsis thaliana*	Positive regulator of heat and drought stress	[115]
VvNAC72	XM_002284632	*Vitis vinifera*	Positive regulator of resistance to pathogen stress	[120]

Supplementary Figure S1. Conserved MEME motifs and their putative correspondence with the A–E subdomains of the NAC domain in FaNAC proteins. The schematic shows the most frequently observed organization of MEME-identified motifs within the NAC domain. Motif correspondence with the A–E subdomains was determined by comparison of their consensus sequences with the NAC domain architecture described by Ooka et al. [78]. Motif frequency is shown as the number of FaNAC proteins containing each motif relative to the total number analysed in this study (n = 33). Motifs 8 and 10 were not assigned to the A–E subdomains.

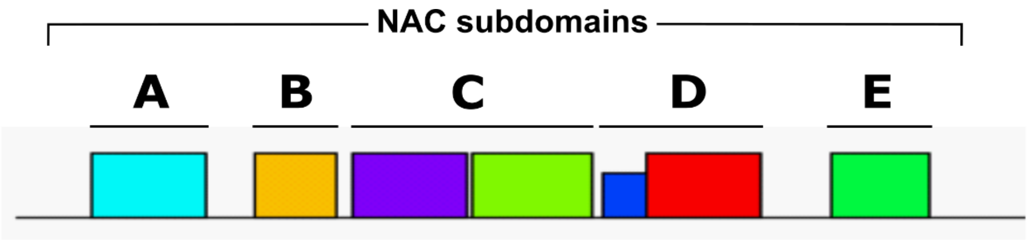

Motif	Symbol	Motif sequence consensus (MEME)	Putative NAC subdomain [78]	Frequency
1		LVFYKGRAPKGEKTBWIMHEY	D	30/33 (90,9%)
2		PPGFRFHPTDEELVSYYLKKK	A	33/33 (100%)
3		RTNRATGSGYWKATGKDKPIYS	C	32/33 (96,9%)
4		AKSGEKEWYFFSPRDRKYPNG	C	29/33 (87,8%)
5		IIAEIDJYKYDPWDL	B	31/33 (93,9%)
6		KDDWVLCRIFKKSGSSKK	E	32/33 (96,9%)
7		RVVGMKKT	D	29/33 (87,8%)
8		FIPTJEGENGICYTHPEKLPGVNKEG QIRHFFHRPSKAYTTGSRKRRKVH	Not asignated	2/33 (6,06%)
9		HLGNNEEEKDGELVLSKVFYQTQPRQCG	E	1/33 (3,03%)
10		QYGAPFKEEDWDDD	Not asignated	4/33 (12,1%)

Supplementary Figure S2. Quantification by RT-qPCR of the relative expression levels of the *FaNAC6* gene in the *Nicotiana benthamiana* transgenic lines (*FaNAC6*-OX) generated. The yellow arrows indicate the transgenic lines selected for study.

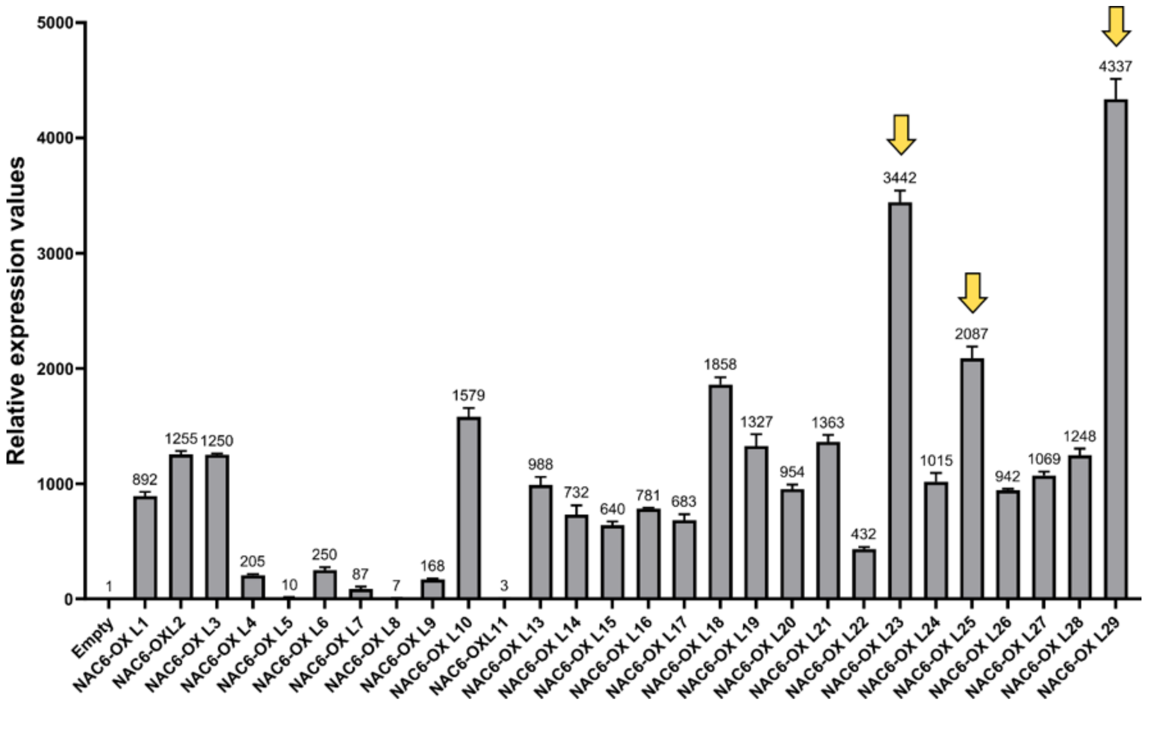

Supplement: Supplementary file 1 [file ijms-27-07352-s001.zip › Supplementary material -NAC6.pdf]
